# Proteome adaptations of the organohalide-respiring *Desulfitobacterium hafniense* strain DCB-2 to various energy metabolisms

**DOI:** 10.3389/fmicb.2023.1058127

**Published:** 2023-01-17

**Authors:** Mathilde Stéphanie Willemin, Romain Hamelin, Florence Armand, Christof Holliger, Julien Maillard

**Affiliations:** ^1^Laboratory for Environmental Biotechnology (LBE), Ecole Polytechnique Fédérale de Lausanne (EPFL), Lausanne, Switzerland; ^2^Proteomic Core Facility (PCF), Ecole Polytechnique Fédérale de Lausanne (EPFL), Lausanne, Switzerland

**Keywords:** *Desulfitobacterium hafniense* strain DCB-2, energy metabolism, organohalide respiration, fermentation, comparative proteomics, Tandem Mass Tag labelling

## Abstract

**Introduction:**

*Desulfitobacterium hafniense* was isolated for its ability to use organohalogens as terminal electron acceptors *via* organohalide respiration (OHR). In contrast to obligate OHR bacteria, *Desulfitobacterium* spp. show a highly versatile energy metabolism with the capacity to use different electron donors and acceptors and to grow fermentatively. *Desulfitobacterium* genomes display numerous and apparently redundant members of redox enzyme families which confirm their metabolic potential. Nonetheless, the enzymes responsible for many metabolic traits are not yet identified.

**Methods:**

In the present work, we conducted an extended proteomic study by comparing the proteomes of *Desulfitobacterium hafniense* strain DCB-2 cultivated in combinations of electron donors and acceptors, triggering five alternative respiratory metabolisms that include OHR, as well as fermentation. Tandem Mass Tag labelling proteomics allowed us to identify and quantify almost 60% of the predicted proteome of strain DCB-2 (2,796 proteins) in all six growth conditions. Raw data are available *via* ProteomeXchange with identifier PXD030393.

**Results and discussion:**

This dataset was analyzed in order to highlight the proteins that were significantly up-regulated in one or a subset of growth conditions and to identify possible key players in the different energy metabolisms. The addition of sodium sulfide as reducing agent in the medium – a very widespread practice in the cultivation of strictly anaerobic bacteria – triggered the expression of the dissimilatory sulfite reduction pathway in relatively less favorable conditions such as fermentative growth on pyruvate, respiration with H_2_ as electron donor and OHR conditions. The presence of H_2_, CO_2_ and acetate in the medium induced several metabolic pathways involved in carbon metabolism including the Wood-Ljungdahl pathway and two pathways related to the fermentation of butyrate that rely on electron-bifurcating enzymes. While the predicted fumarate reductase appears to be constitutively expressed, a new lactate dehydrogenase and lactate transporters were identified. Finally, the OHR metabolism with 3-chloro-4-hydroxyphenylacetate as electron acceptor strongly induced proteins encoded in several reductive dehalogenase gene clusters, as well as four new proteins related to corrinoid metabolism. We believe that this extended proteomic database represents a new landmark in understanding the metabolic versatility of *Desulfitobacterium* spp. and provides a solid basis for addressing future research questions.

## 1. Introduction

Numerous studies focusing on differential gene expression in organohalide-respiring bacteria (OHRB) have been published over the years. Both transcriptomic and proteomic approaches contributed to today’s comprehension of the OHR metabolism. Global proteome analyses were performed with representative bacteria from all OHRB phyla but the level of proteome coverage achieved varies significantly from one study to the other (Türkowsky et al., 2018). Globally, the available datasets are largely dominated by studies targeting *Dehalococcoides mccartyi* spp. which somehow imbalance the global understanding of the OHR process. Only a few studies analyzing the proteomes of facultative OHRB such as *Sulfurospirillum multivorans* (Goris et al., 2015) or *Desulfitobacterium dehalogenans* (Kruse et al., 2015) growing on different substrates have been published.

Based on genomic data, *Desulfitobacterium* spp. display a fairly complex phylogeny with relatively high genetic redundancy within individual genomes and an important metabolic diversity across the isolated species and strains, features that have been well documented (Villemur et al., 2006; Kruse et al., 2017). *Desulfitobacterium hafniense* strain DCB-2 was the first member of the *Desulfitobacterium* genus to be isolated (Madsen and Licht, 1992) and also the first to be sequenced (Kim et al., 2012). Among facultative OHRB, strain DCB-2 displays the highest number of reductive dehalogenase gene clusters (Kruse et al., 2017) and thus represents an excellent candidate for studying its energy metabolism in general and the adaptations to OHR metabolism. Twelve years ago, we published the first comparative proteomic analysis on a member of the energetically versatile *Desulfitobacterium* genus (Prat et al., 2011). Despite the low resolution of the proteomic data that was obtained from 2D gel electrophoresis, it revealed that *Desulfitobacterium hafniense* strain TCE1 adapted its proteome to the presence of tetrachloroethene (PCE) by inducing proteins involved in the stress response (CodY, PspA, GroEL). PceA, the key enzyme in PCE reductive dechlorination was also up-regulated when compared to cells cultivated with fumarate as alternative electron acceptor. It was shown, however, that it was not due to a dedicated regulatory pathway but to the instability of the *pce* gene cluster in strain TCE1 (Duret et al., 2012). Strikingly also, the Wood-Ljungdahl pathway (WLP) was clearly up-regulated in PCE conditions, however only when hydrogen and acetate were used as electron donor and carbon source, respectively. The OHR pathway has been also investigated by comparative proteomics in *Desulfitobacterium dehalogenans* strain JW/IU_DC1^T^ (Kruse et al., 2015). The strain was cultivated either with pyruvate only (fermentative growth), or with combinations of formate and fumarate, or with formate and 3-chloro-4-hydroxyphenylacetate (ClOHPA), the latter triggering OHR metabolism. While CprA [also named as RdhA6 (Kim et al., 2012)], the terminal reductase in OHR, was strongly up-regulated in presence of ClOHPA, new possible protein candidates have been proposed to be part of the electron transfer chain between the menaquinone pool and CprA. Although no biochemical evidence has been obtained, a *c*-type cytochrome (NrfAH) and two flavoproteins (Desde_3368 and _3673) have been considered as possibly involved in OHR, with Desde_3368 being largely up-regulated during ClOHPA respiration. This protein could act as an extracytoplasmic electron shuttle between a yet unknow quinol-oxidizing membrane-bound complex and CprA (Kruse et al., 2015). An extensive study has investigated the metabolic flexibility of *Desulfitobacterium hafniense* strain Y51 under electron donor (lactate) and electron acceptor (fumarate) limitations in chemostats (Marozava et al., 2018). Surprisingly, the excess of lactate (under fumarate limitation) and of fumarate (under lactate limitation) were consumed in chemostat, indicating a possible lactate fermentation and fumarate disproportionation allowing strain Y51 to grow in absence of the electron acceptor or donor, respectively. Proteomic analysis has suggested that alcoholic fermentation, sulfur metabolism and the Wood-Ljungdahl pathway are possible alternative energy metabolisms under electron donor and acceptor limitations. The study has also shown discrepancies in the regulation patterns between transcriptional and translational levels, thus arguing in favor of proteomics as a more direct tool than transcriptomics to assess metabolic activities. Nevertheless, the proteome coverage of the studies by Kruse et al. and Marozava et al. were either limited or only high in one or the other growth conditions impairing an extended proteome comparison.

In the present work, a Tandem Mass Tag (TMT) based quantitative proteomic approach was used to measure the relative abundance of proteins in *Desulfitobacterium hafniense* strain DCB-2 that was cultivated in six different growth conditions. The growth conditions were defined by combinations of electron donors and acceptors to study proteome adaptations in this energetically versatile OHRB. On the one hand, the well-studied pyruvate and fumarate respiration was compared to pyruvate-only which is considered as fermentative growth, and, on the other hand the alternative use of lactate or H_2_ as electron donors in combination with either fumarate or 3-chloro-4-hydroxyphenylacetate (ClOHPA) as electron acceptors help identifying substrate-specific metabolic pathways.

## 2. Materials and methods

All chemicals used in the following protocols were purchased at Merck (Coinsins, Switzerland) unless specified otherwise.

### 2.1. Cultivation of *Desulfitobacterium hafniense* strain DCB-2

*Desulfitobacterium hafniense* strain DCB-2 (DSM 10664) was cultivated anaerobically in 40-ml or 200-ml liquid batch cultures. The growth medium consisted of a basal solution containing the following major elements: 0.958 g·l^−1^ of K_2_HPO_4_·3 H_2_O, 0.218 g·l^−1^ of NaH_2_PO_4_·2 H_2_O, 0.05 g·l^−1^ of yeast extract and 0.05 g·l^−1^ of resazurin as redox indicator. The basal solution was supplemented with two other solutions prepared separately which were both added at a 1:40 (v/v) ratio. The first one consisted of vitamins and trace elements (20 mg·l^−1^ of EDTA (pH = 7.0), 80 mg·l^−1^ of FeCl_2_·4 H_2_O, 4 mg·l^−1^ of MnCl_2_·4 H_2_O, 7.6 mg·l^−1^ of CoCl_2_·6 H_2_O, 2.8 mg·l^−1^ of ZnCl_2_, 0.102 mg·l^−1^ of CuCl_2_·2 H_2_O, 0.2208 mg·l^−1^ of AlCl_3_, 0.24 mg·l^−1^ of H_3_BO_3_, 1.656 mg·l^−1^ of Na_2_MoO_4_·2 H_2_O, 0.96 mg·l^−1^ of NiCl_2_ ·6 H_2_O, 2 mg·l^−1^ of biotin, 10 mg·l^−1^ of *p*-aminobenzoate, 2 mg·l^−1^ of pantothenate, 0.8 mg·l^−1^ of folic acid (2 H_2_O), 2 mg·l^−1^ of lipoic acid, 0.4 mg·l^−1^ of pyridoxine, 22 mg·l^−1^ of nicotinic acid, 4 mg·l^−1^ of thiamine-HCl, 2 mg·l^−1^ of riboflavin and 2mg. l^−1^ of cyanocobalamin). The second solution was prepared by dissolving 4.4 g·l^−1^ of CaCl_2_ 2 H_2_O and 4.06 g·l^−1^ of MgCl_2_·6H_2_O in ddH2O. Finally, a third solution of concentrated carbonate/bicarbonate buffer (9.01 g·l^−1^ of NH_4_HCO_3_ and 76.11 g·l^−1^ NaHCO_3_) supplemented with 0.48 g·l^−1^ of Na_2_S·9 H_2_O was added at 1:20 (v/v) ratio to the growth medium. All the separate solutions were made anaerobic through 15 cycles of gas-exchange with N_2_, (or N_2_/CO_2_ at 80%/20% for the carbonate/bicarbonate buffer), sterilized either by autoclave or filtration and stored at 4°C. Prior to inoculation, the medium was supplemented with different combinations of electron donors and acceptors in order to trigger different types of metabolism. For triggering fermentative metabolism, the medium was supplemented with a final concentration of 40 mM of pyruvate (Py-only). Alternatively, pyruvate was also used in respiratory metabolism as electron donor at a final concentration of 20 mM in combination with 20 mM of fumarate as electron acceptor (Py/Fu). Pyruvate was replaced with 20 mM of lactate (La/Fu). To trigger OHR metabolism, 3-chloro-4-hydroxyphenylacetic acid (ClOHPA) was used as electron acceptor at a final concentration 10 mM (La/ClOHPA). In addition, molecular hydrogen was used as electron donor by replacing the gas phase with a mixture of 80% H_2_/20% CO_2_, either in combination with fumarate (H_2_/Fu) or ClOHPA (H_2_/ClOHPA). In the case of H_2_, 2 mM of acetic acid was added as the carbon source. The culture flasks used for proteomic analysis were inoculated with 5% (v/v) of actively growing cultures after at least three successive transfers in the same growth conditions. All cultures were incubated in the dark at 30°C under agitation on an orbital shaker at 100 rpm.

The effect of the addition of 1 mM of Na_2_S as reducing agent in the anaerobic medium was assessed by performing triplicate 200-ml cultures with pyruvate as carbon and energy source (Py-only, as above) in presence or absence of Na_2_S in the medium. When Na_2_S was omitted, in order to render the medium fully anaerobic 20 μl of a Titanium(III)-citrate stock solution (1.5% Titanium(III) chloride in 0.2 M citrate, pH 8.0) was added to 200 ml of medium. One additional series of triplicate cultures was performed in Py-only devoid of Na_2_S but supplemented with 1 mM sodium sulfite.

### 2.2. Culture monitoring and analytical methods

The cultures were monitored for growth by measuring the absorbance at 600 nm (A_600_) and the concentration of total proteins using the Qubit™ Protein Assay Kit (ThermoFisher Scientific). For the experiment for testing the effect of Na_2_S, the concentrations of pyruvate, lactate, and acetate were determined by HPLC from 1.5-ml filtered culture aliquots as described previously (Weissbrodt et al., 2012).

For the investigation of sulfur-related genes, 1 mM of Na_2_S was spiked into duplicate 40-ml cultures of strain DCB-2 after 20 h of growth in Py-only conditions along with duplicate non-spiked cultures. After 5 h of incubation, the biomass was collected for RNA extraction, reverse transcription and quantitative PCR as previously described (Cimmino et al., 2022). Selected genes involved in sulfur metabolism were targeted with specific primer sets (Supplementary Table 1).

Batch cultures of 40-ml were also performed to assess the protein profiles in SDS-PAGE from cells cultivated in Py-only in presence or absence of Na_2_S as reducing agent (as above), and in three other conditions devoid of Na_2_S: pyruvate +1 mM sodium sulfite (PySu_1_), lactate +5 mM sodium sulfite (La/Su_5_) and lactate +10 mM isethionate. The cultures from the third transfer was collected by 20 min centrifugation at 4500× *g* and 4°C, washed twice in 50 mM of Tris–HCl buffer (pH 7.5), resuspended in 400 μl of lysis buffer (Tris buffer supplemented with protease inhibitors as above). The cell suspensions were lysed by sonication (as indicated above) and centrifugated for 5 min at 500× *g* to remove unbroken cells. The protein concentration of cell-free extracts was measured with the BCA assay and 20 μg of proteins were loaded as a 12.5% acrylamide gel. SDS-PAGE was and gel staining with Coomassie were performed according to standard procedures.

### 2.3. Cell harvesting and sample preparation for proteomic analysis

The biomass of triplicate batch cultures for each growth condition (18 samples in total) was collected in the rather late growth phase to maximize the amount of biomass produced and analyze proteomes of physiologically active cells. The biomass was pelleted for 15 min at 4500× *g* and 4°C. Biomass pellets were washed twice in ice-cold 50 mM Tris–HCl buffer (pH 7.5) and stored in 1.5-ml Eppendorf tubes at-80°C until further use. All biomass samples used for proteomic analysis were treated at the same time. First, the pellets were resuspended in 100 μl of 50 mM HEPES buffer (pH 8.0) supplemented with SigmaFast™ protease inhibitor cocktail (Sigma Aldrich, Buchs, Switzerland) and a few crystals of DNase I (Roche, Basel, Switzerland). Five rounds of 5 × 1 s of sonication at 60% amplitude were applied to lyse the cells on ice (Fisherbrand™ Q500 sonicator, Thermo Fisher Scientific, Basel, Switzerland). After a soft centrifugation (500× *g*, 5 min and 4°C) to remove unbroken cells, proteins from cell-free extracts were solubilized for 15 min in 2% SDS at room temperature. Protein concentration was determined using Pierce™ BCA protein assay kit (Fisher Scientific) and protein samples were diluted with 2% SDS solution and aliquoted to 30 μg in 30 μl. Samples were stored at-80°C until proteomic analysis.

### 2.4. Proteomic analysis

#### 2.4.1. Reduction/alkylation of the samples

Samples were trypsin digested using the Filter-Aided Sample Preparation (FASP) protocol with minor modifications (Wiśniewski et al., 2009). SDS-treated protein samples of 30 μg were resuspended in 200 μl of 8 M urea solution in 100 mM Tris–HCl and deposited on top of Microcon®-30 K devices (Merck AG, Zug, Switzerland). Samples were centrifuged at 9400× *g*, at 20°C for 30 min. All subsequent centrifugation steps were performed using the same conditions. An additional 200 μl of 8 M urea solution was added and devices were centrifuged again. Reduction was performed by adding 100 μl of 10 mM Tris(2-carboxy)phosphine (TCEP) in 8 M urea solution on top of filters followed by 60 min incubation time at 37°C with gentle shaking and light protection. Reduction solution was removed by centrifugation and filters were washed with 200 μl of 8 M urea solution. After removal of washing solution by centrifugation, alkylation was performed by adding 100 μl of 40 mM chloroacetamide in 8 M urea solution and incubating the filters at 37°C for 45 min with gentle shaking and protection from light. The alkylation solution was removed by centrifugation and another washing/centrifugation step with 200 μl of 8 M urea solution was performed. This last urea buffer washing step was repeated twice followed by three additional washing steps with 100 μl of 5 mM Tris–HCl. Proteolytic digestion was performed overnight at 37°C by adding on top of filters 100 μl of a combined solution of Endoproteinase Lys-C and Trypsin Gold in an enzyme/protein ratio of 1:50 (w/w) supplemented with 10 mM CaCl_2_. Resulting peptides were recovered by centrifugation. The devices were then rinsed with 50 μl of 4% trifluoroacetic acid and centrifuged. This step was repeated three times and peptides were finally desalted on SDB-RPS StageTips (Kulak et al., 2014) and dried by vacuum centrifugation. A mixture of each biological samples was spiked as two additional channels and used as bridge channels.

#### 2.4.2. TMT-labeling

For Tandem Mass Tag (TMT) labelling, dried peptides were first reconstituted in 8 μl of 100 mM HEPES pH 8 and 3 μl of TMT solution (40 μg·μl^−1^ in pure acetonitrile) was then added. TMT Labelling was performed with the TMT10plex™ isobaric Mass Tagging Kit (ThermoFisher Scientific) at room temperature for 90 min and reactions were quenched with hydroxylamine to a final concentration of 0.4% (v/v) for 15 min. TMT-labelled samples were then pooled at a 1:1 ratio across all samples. A single shot LC–MS control run was performed to ensure similar peptide mixing across each TMT channel to avoid the need of further excessive normalization. Quantities of each TMT-labelled sample were adjusted according to the control run. The combined samples were then desalted using a 100 mg Sep-Pak C18 cartridge (Waters AG, Baden-Dättwil, Switzerland) and vacuum centrifuged. Pooled samples were fractionated into 12 fractions using an Agilent OFF-Gel 3,100 system following the manufacturer’s instructions. Resulting fractions were dried by vacuum centrifugation and again desalted on C18 StageTips.

#### 2.4.3. LC–MS/MS analysis

Each individual fraction was resuspended in 2% acetonitrile in 0.1% formic acid and nano-flow separations were performed on a Dionex Ultimate 3,000 RSLC nano UPLC system on-line connected with a Lumos Fusion Orbitrap Mass Spectrometer. A capillary pre-column (Acclaim Pepmap C18; 3 μm-100 Å; 2 cm x 75 μM ID) was used for sample trapping and cleaning. Analytical separations were performed at 250 nl/min over a 150 min biphasic gradient on a 50 cm long in-house packed capillary column (75 μm ID, ReproSil-Pur C18-AQ 1.9 μm silica beads; Dr. Maisch). Acquisitions were performed through Top Speed Data-Dependent acquisition mode using 3 s cycle time. First MS scans were acquired at a resolution of 120′000 (at 200 m/z) and the most intense parent ions were selected and fragmented by High energy Collision Dissociation (HCD) with a Normalized Collision Energy (NCE) of 37.5% using an isolation window of 0.7 m/z. Fragmented ions scans were acquired with a resolution of 50′000 (at 200 m/z) and selected ions were then excluded for the following 120 s.

#### 2.4.4. Data analysis

Raw data were processed using SEQUEST, Mascot, MS Amanda (Kulak et al., 2014) and MS Fragger (Dorfer et al., 2014) in Proteome Discoverer v.2.4 against the *Desulfitobacterium hafniense* strain DCB-2 database (4,851 protein sequences). Enzyme specificity was set to trypsin and a minimum of six amino acids was required for peptide identification. Up to two missed cleavages were allowed and a 1% false discovery rate (FDR) cut-off was applied both at peptide and protein identification levels. For the database search, carbamidomethylation (C), TMT tags (K and peptide N termini) were set as fixed modifications whereas oxidation (M) was considered as a variable one. Resulting text files were processed through in-house written R scripts (version 3.6.3) (Kong et al., 2017). A first normalization step was applied according to the Sample Loading normalization (R Core Team, 2018). Assuming that the total protein abundances were equal across the TMT channels, the reporter ion intensities of all spectra were summed and each channel was scaled according to this sum, so that the sum of reporter ion signals per channel equals the average of the signals across samples. The multiplexing design of the experiment required a second normalization step to correct variations between the two TMT experiments. Internal Reference Scaling (IRS) process was here applied (Plubell et al., 2017). For each TMT run, the mean of reporter ion signals in the two corresponding bridge channels was calculated for each protein. The normalization factor, scaling the two bridge channels means between the two TMT experiments, was used to scale protein intensities. A Trimmed M-Mean normalization step was also applied using the package EdgeR (version 3.26.8; Robinson et al., 2010). Assuming that samples contain a majority of non-differentially expressed proteins, this third step calculates normalization factors according to these presumed unchanged protein abundances. Proteins with high or low abundances and proteins with larger or smaller fold-changes were not considered. Proteins that were quantified in the 22 channels across the two TMT batches were used for the following data treatments. Differential protein expression analysis was performed using R bioconductor package limma (version 3.34.9, 2018-02-22; Ritchie et al., 2015), followed by the Benjamini-Hochberg procedure (Benjamini and Hochberg, 1995). Normalized and standardized protein abundances were used for the following data treatments. A heat map was processed to visualize unsupervised hierarchical clustering of protein abundances using the Euclidean distance, expressed as *Z*-scores. The *Z*-scores indicate if the protein is more (positive scores) or less (negative scores) expressed across the different samples in comparison to its mean abundance.

### 2.5. Proteomic data accessibility

The mass spectrometry proteomics data have been deposited to the ProteomeXchange Consortium *via* the PRIDE (Perez-Riverol et al., 2019) partner repository with the dataset identifier PXD030393.^1^

## 3. Results and discussion

In the present study, the proteomes of cells of *Desulfitobacterium hafniense* strain DCB-2 cultivated in six different growth conditions were compared. Fermentative growth was targeted by the addition of pyruvate as sole carbon and energy substrate. The five other conditions combined one electron donor and one electron acceptor, thus favoring respiratory metabolisms, including OHR. While a small amount of yeast extract was added to all the media, its effect on the physiology and proteome of strain DCB-2 was considered as negligible. Growth of strain DCB-2 in these conditions was monitored in another set of cultures. The growth performances of strain DCB-2 varied substantially depending on the substrates used (Supplementary Figure 1), ranging in maximal absorbance (A_600_) values from 0.08 (cultures with H_2_ as electron donor) to 0.45 (Py/Fu). The 18 samples dedicated to proteomics were split into two independent runs of TMT labelling (each with 10 different isobaric mass tags). One tag per run was used to label the mixture of all 18 samples which served in the normalization procedure as presented in Material and Methods.

### 3.1. General output of the proteomic analysis

A total of 2,936 proteins were detected and 2,796 among them were quantified across all of the 18 samples analyzed (Supplementary Table 2)The latter number represents 58% of the theoretical proteome of *Desulfitobacterium hafniense* strain DCB-2 (Kim et al., 2012). Thus, in OHRB the present study resulted in the most extended proteomic dataset so far obtained (in the absolute number of proteins). Our dataset goes beyond the 1,023 proteins quantified in *Dehalococcoides mccartyi* (Schiffmann et al., 2016) and the 1,055 proteins identified in *Dehalobacter restrictus* strain PER-K23 (Rupakula et al., 2013), representing 66 and 33% of their proteome, respectively. Furthermore, our study describes the proteome of strain DCB-2 in six different growth conditions. In comparison to previous proteomic studies on *Desulfitobacterium* spp. (Prat et al., 2011; Kruse et al., 2015; Marozava et al., 2018), our comparative proteomic dataset clearly improved the resolution of their rather large proteomes, both in the number of identified proteins and in the number of pairwise growth condition comparisons. After several steps of normalization, a principal component analysis (PCA) with the data of the replicate batch cultures revealed a satisfying reproducibility (Supplementary Figure 2). Moreover, the two axes of the PCA display 57% of the sample variability centered around the artificially mixed samples, which reflected the consistency of both TMT runs. The PCA revealed mainly two subgroups of growth conditions with Py/Fu and La/Fu on one side and the four other conditions on the other side of the major axis (PC1). The second axis (PC2) shows that the use of H_2_ as electron donor is the major driver for the variability in the proteome of strain DCB-2. The use of pyruvate as sole source of carbon and energy (Py-only) also seemed to be discriminating in comparison to respiratory metabolisms. This sample variability and distribution were confirmed by the hierarchical clustering of the proteomes that was obtained from Z-scores of the normalized abundances of the proteins quantified in the different growth conditions (Supplementary Figure 3). All the quantified proteins displaying logFC values <−1 and > 1 and an FDR value <0.05 in at least one pairwise comparison (i.e., 1,005 proteins) were grouped into 9 clusters as shown in the heatmap according to their relative abundance, thus helping to identify proteins involved in specific metabolic pathways (Supplementary Figure 3; Supplementary Table 3).

The comparative proteomic analysis gave rise to a large amount of data. Specific multiple pairwise comparisons of the proteomic dataset were selected to investigate the following proteome adaptations of *Desulfitobacterium hafniense* strain DCB-2: fermentative vs. respiratory metabolisms; influence of the electron donor (lactate vs. H_2_); and influence of the electron acceptor (fumarate vs. ClOHPA).

### 3.2. Proteome adaptations to partial fermentative growth conditions

**Figure 1:**
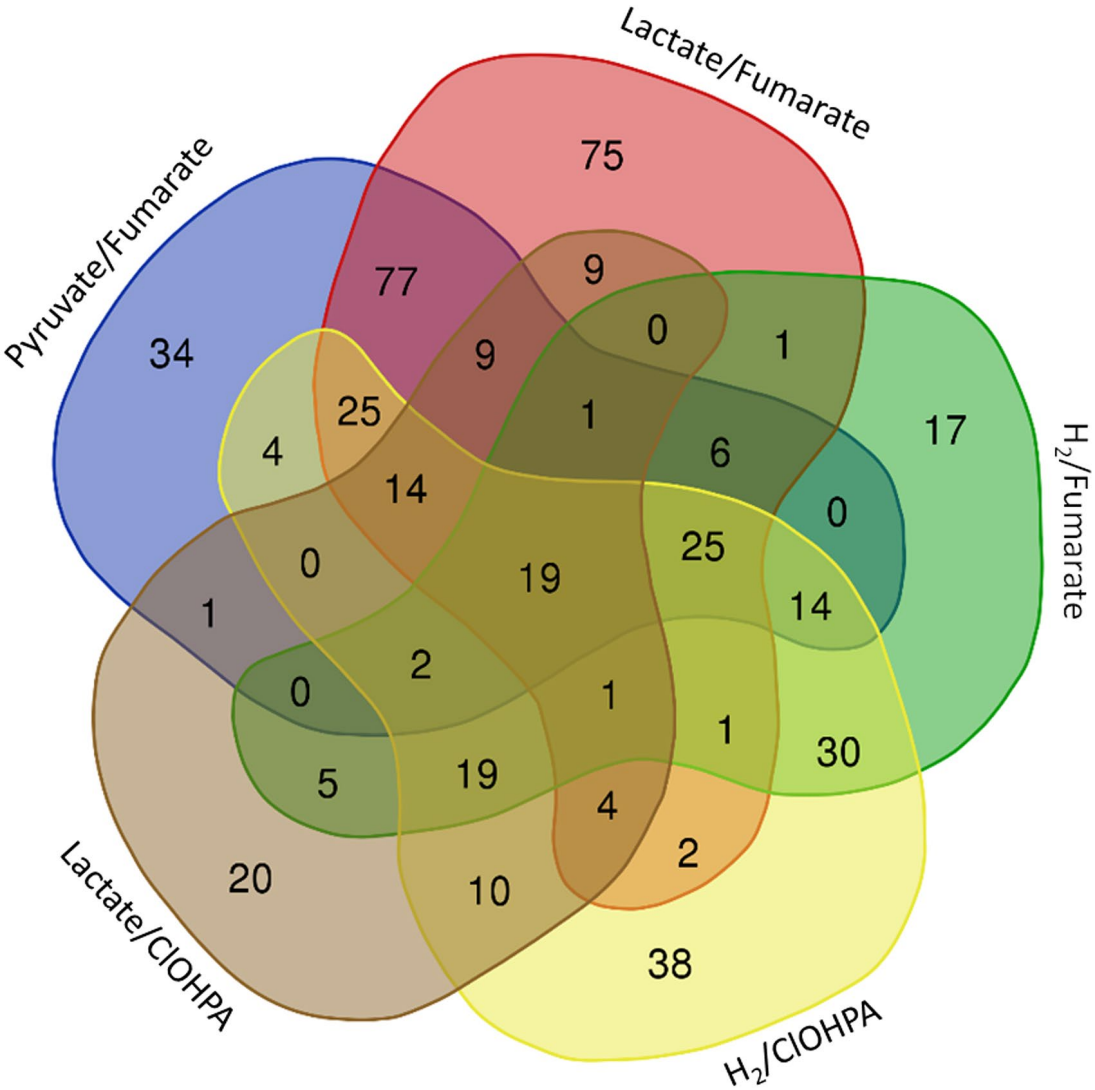
Venn diagram representing the number and distribution of 463 proteins identified as significantly up-regulated in fermentative growth conditions (Py-only) in comparison to any of the respiratory metabolisms (see also Supplementary Table 4). The 19 proteins at the centre of the diagram are listed in Table 1.

The proteomic dataset was first analyzed with emphasis on the proteins showing significant levels of increased relative abundance (in short “up-regulated proteins”; this well-accepted term is used throughout the paper) during growth with pyruvate as sole energy and carbon source (Py-only) in comparison to other conditions, i.e., all combinations of electron donor and acceptor. As displayed in Figure 1, a selection of 463 proteins displayed at least a two-fold significant up-regulation (logFC >1) in Py-only against any of the respiratory conditions (Supplementary Table 4), with a set of 19 proteins systematically up-regulated in comparison to all of the other growth conditions (Table 1). While the 463 proteins are widely distributed across several clusters, most of the 19 proteins were found in cluster 6 (Supplementary Table 3). The corresponding heatmap shows a distinct pattern in cluster 6 for most of these proteins (top branch in Supplementary Figure 3), highlighting their homogenous distribution in the proteomic dataset. In this section are discussed the identification of the isethionate metabolizing proteins, an unusual composite NADP-dependent hydrogenase, and a carbon monoxide dehydrogenase. Most importantly here, the up-regulation of the dissimilatory sulfite reduction pathway led us to reconsider the energy metabolism of cells growing with addition of pyruvate only as a partial fermentative route (See also section 1.1.1 in Supplementary material for additional findings identified in the proteome of cells growing on pyruvate only.)

**Table 1:** Proteins significantly up-regulated in fermentative conditions (Py-only) vs. all respiratory conditions.

Accession	Predicted function	Fold-change in Py-only vs.				
		Py/Fu	La/Fu	H_2_/Fu	H_2_/Cl	La/Cl
ACL18433	Glycyl radical enzyme of unknown function (GUF)	8	5	4	9	3
ACL18746	Succinate:quinone oxidoreductase, flavoprotein	7	4	9	9	4
ACL19672	Transcriptional regulator, CdaR family	2	2	2	2	2
ACL20074	NADP-oxidoreductase/NADPH-reducing hydrogenase	8	4	6	6	3
ACL20535	Succinate:quinone oxidoreductase, flavoprotein	17	5	9	11	4
ACL21007	Succinate:quinone oxidoreductase, flavoprotein	6	5	5	3	2
ACL21108	NAD-dependent decarboxylating malate DH	30	8	10	9	9
ACL21109	Citryl-CoA lyase, β subunit	22	7	14	11	6
ACL21110	Succinyl-CoA synthetase, α subunit	13	6	11	10	7
ACL21111	Hypothetical protein	11	5	9	9	5
ACL21112	Succinyl-CoA synthetase, β subunit	15	5	11	9	7
ACL21807	4Fe-4S ferredoxin	3	4	16	16	12
ACL21808	CODH, clade F, catalytic subunit	3	5	22	20	15
ACL21809	Cobyrinic acid *a,c*-diamide synthase	3	3	3	3	3
ACL22391	Transcriptional regulator, IclR family	3	3	3	2	3
ACL22517	Methyl-accepting chemotaxis sensory transducer	5	4	5	3	7
ACL22518	Protein of unknown function (DUF224)	8	4	8	5	17
ACL22519	FAD-linked oxidase protein	9	5	8	5	25
ACL22584	ABC transporter	5	4	2	3	4

#### 3.2.1. Identification of the isethionate metabolising protein

A glycyl radical enzyme (GRE), ACL18433, was found significantly up-regulated between 3× and 9× fold-change in Py-only conditions. GREs are diverse in function but can be classified into two groups based on the generation of aldehydes (or lack thereof). GREs generating aldehydes require the establishment of bacterial micro-compartments (BMC), protein-based organelles that protect the cytoplasm from toxic reaction intermediates (Kerfeld et al., 2018). Initially, Zarzycki et al. have identified ACL18433 as a GRE of unknown function (GUF) based on the peculiar gene composition and organization (Zarzycki et al., 2015). More recently, Xing et al. have clearly identified the GUF protein (incl. homologous proteins in *Desulfitobacterium* spp.) as isethionate sulfo-lyase, consisting of IseG and IseH, and involved in C2 sulfonate degradation in anaerobic bacteria (Xing et al., 2019). ACL18433 (the gene encoding IseG) is likely part of a five-gene operon (ACL18432-6), from which three proteins were detected as significantly up-regulated during fermentation (Supplementary Table 4). While ACL18432 is a transcriptional regulator homologous to IseK from *Desulfovibrio piger* (Xing et al., 2019), ACL18434 is the GRE-associated activating enzyme IseH (Shisler and Broderick, 2014). ACL18435 and ACL18436 belong to the family of tripartite ATP-independent periplasmic (TRAP) transporters (Forward et al., 1997) that is composed of two subunits homologous to DctP and to a fusion of DctQ and DctM (Mulligan et al., 2011), and which is likely involved in the uptake of sulfonates. Interestingly, genes coding for bacterial micro-compartment (BMC) proteins as well as an acetaldehyde dehydrogenase (ACL18423) and for proteins homologous to subunits of the ethanolamine or propanediol utilization enzymes are located directly upstream of the ACL18432-6 gene cluster (Supplementary Figure 4A). As no isethionate is present in the culture medium, it is not yet clear why these proteins are up-regulated in Py-only condition, but it may be related to the presence of sodium sulfide that was used as reducing agent and to the generation of oxidized sulfur derivatives in the culture medium. Nevertheless, the protein pattern obtained from cells growing on isethionate harbors at least one specific band below the 100 kDa marker that is likely to be ACL18433 (IseG) (indicated by a star in Supplementary Figure 4B). LC–MS/MS analysis of the corresponding gel piece unambiguously confirmed the presence of IseG as it was detected with the highest relative abundance and a protein coverage of 94% (Supplementary Figure 4C). Overall, this confirms and extends the early work on *Desulfitobacterium* and isethionate (Lie et al., 1999).

#### 3.2.2. An unusual composite NADP-dependent hydrogenase

From the list of the 19 proteins systematically up-regulated in Py-only conditions, ACL20074 was found significantly up-regulated with 3–8× fold-change. This large protein, initially annotated as Fe-only hydrogenase in strain DCB-2, is worth mentioning since it appears as a patchwork of different redox enzymes when compared to proteins from the UniProt database. The major part of the N-terminal half of ACL20074, with exception of the first 110 amino acids, looks like an NADPH-dependent oxidoreductase, with SfrB of *Geobacter sulfurreducens* as the best match with BlastP (Coppi et al., 2007), while the first 110 amino acids and the C-terminal half resembles to the HndD subunit of the NADP-reducing hydrogenase from *Desulfovibrio fructosovorans* (Malki et al., 1995). SfrAB was proposed to be involved in acetate metabolism with a possible function in the NADPH/NADP homeostasis, while the HndABCD complex has been proposed recently to be a ferredoxin and NAD- (and not NADP-) dependent flavin-based electron-bifurcating hydrogenase (Kpebe et al., 2018). The sequence alignment of ACL20074 with the characterized [FeFe] electron-bifurcating hydrogenases [as taken from (Kpebe et al., 2018)] revealed that the major sequence features of HndD are conserved in ACL20074. Interestingly, the first 110 amino acids of ACL20074, that comprise the conserved [2Fe/2S] cluster, match with the N-terminal sequence of HndD, then the sequence homology stops in the middle of the first [4Fe/4S] cluster and starts again around position 580 of ACL20074. It is the intermediate region of ACL20074 that shows homology to SfrB from *Geobacter sulfurreducens* (Supplementary Figure 5). The lack of the other Hnd subunits in the genetic environment of ACL20074, and most importantly HndC which harbors the flavin-and NAD-binding sites, and is responsible for electron bifurcation, is possibly replaced by the intermediate region in ACL20074 that displays possible binding sites for FAD and NAD(P)H, according to (Kaufmann and Lovley, 2001). Despite the clear sequence homology to the electron-bifurcating hydrogenases and despite the lack of maturation enzymes for the biosynthesis of the [FeFe] cofactor in strain DCB-2 (Kruse et al., 2017), no clear function can be assigned yet to this unusual composite hydrogenase. However, it seems to be widely distributed in the Firmicutes (data not shown) and invites further physiological and biochemical investigations.

#### 3.2.3. Carbon monoxide metabolism by a CO dehydrogenase

One among the four carbon monoxide dehydrogenases (CODH) encoded in the genome of strain DCB-2 was significantly up-regulated in Py-only conditions. ACL21808, the CODH catalytic subunit (CooS), is part of a predicted 3-gene operon (ACL21807-9) that also displays a typical ferredoxin (ACL21807, CooF) and a CODH maturation protein (ACL21809). Sequence analysis revealed that ACL21808 belongs to the clade F1 of CODH enzymes, a clade which also displays the well-characterized monofunctional CODH I and II from *Carboxydothermus hydrogenoformans* (Dobbek et al., 2001; Svetlitchnyi et al., 2001; Inoue et al., 2019). Not seen as up-regulated, ACL21806 (CooA) is a member of the CRP/FNR regulatory protein superfamily and is likely regulating the transcription of the CODH encoding operon. ACL21807-8 homologous proteins in *Desulfitobacterium dehalogenans* (Desde_3263–4) have also been clearly identified as up-regulated in cells growing with pyruvate only (in comparison to formate/fumarate or formate/ClOHPA conditions) without, however, any suggestion of function (Kruse et al., 2015). The exact function ACL21808 is not known, however, its relatively high sequence identity (70%) with the CODH I from *Carboxydothermus hydrogenoformans* may suggest that it appears as a homodimer, that it is associated with the cytoplasmic side of the membrane and is involved in energy conservation by transferring electrons from the oxidation of CO to a H_2_-evolving hydrogenase (CO-driven proton respiration) (Svetlitchnyi et al., 2001). Moreover, in *Rhodospirillum rubrum*, the homologous CODH enzyme was proposed to deliver electrons to a complex I-related membrane-bound Ech hydrogenase (Alfano and Cavazza, 2018). In the case of strain DCB-2, the CODH enzyme could thus transfer electron to the complex I-like enzyme and contribute to proton pumping. However, a pre-requisite for this metabolic activity is the generation of CO that could be possibly derived from the Wood-Ljungdahl pathway (see below for a detail discussion).

#### 3.2.4. The dissimilatory sulfite reduction pathway for extra energy

**Figure 2:**
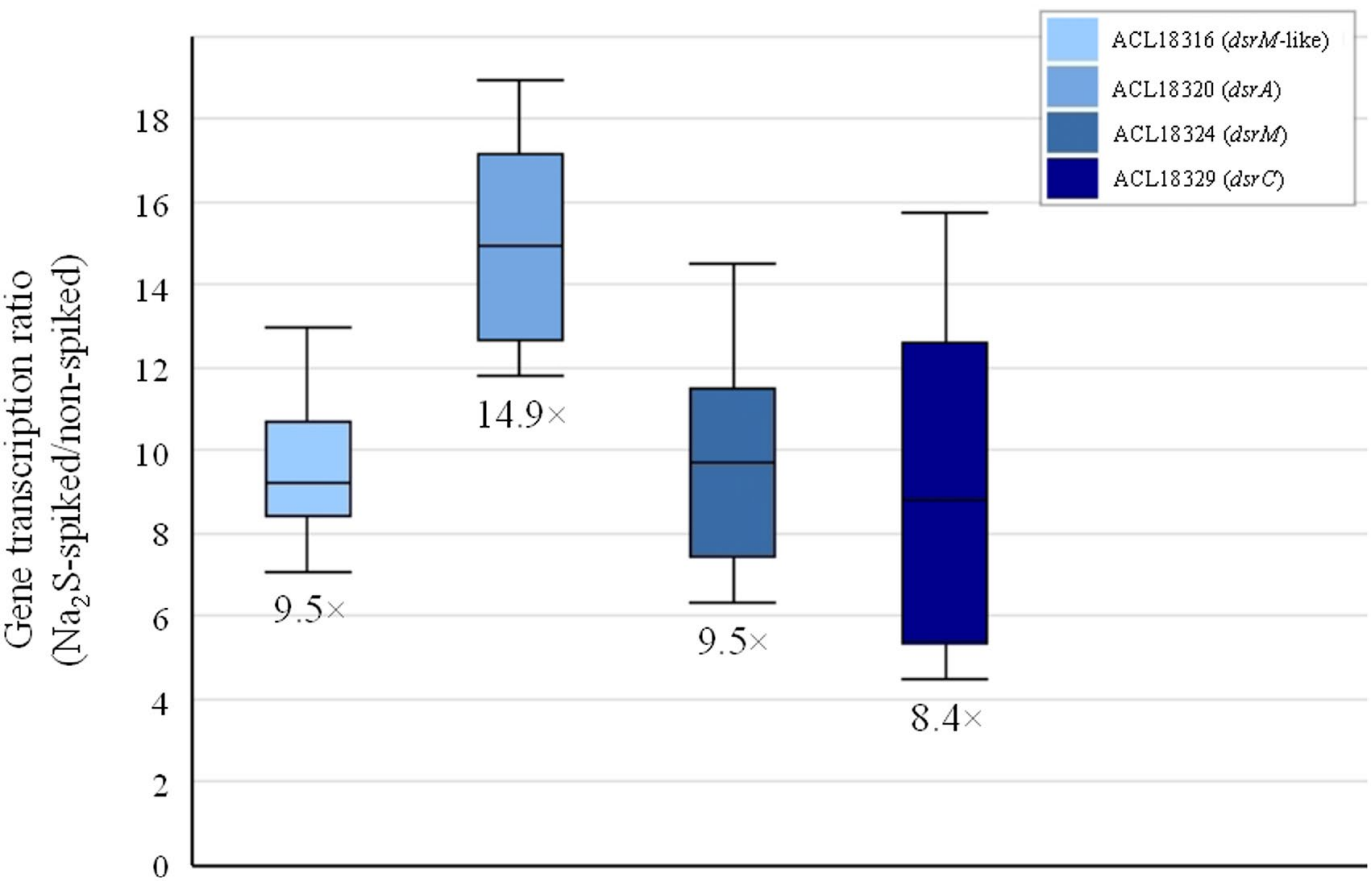
Induction of the transcription of selected *dsr* genes in *Desulfitobacterium hafniense* strain DCB-2 growing on pyruvate upon addition of 1 mM of sodium sulfide.

The direct comparison of the proteome from cells cultivated on pyruvate only with cells growing with pyruvate and fumarate (Py/Fu) revealed 231 proteins that were significantly up-regulated in Py-only conditions (Supplementary Table 4). Among them, a selection of 29 proteins displayed an up-regulation level higher than 10× fold-change (logFC >3.3) and are widespread distributed across several clusters (Supplementary Table 5). Beside the proteins already discussed in Table 1, some proteins are noteworthy. Seven proteins belonging to a 14-gene cluster (ACL18316-29; Supplementary Figure 6A) were also up-regulated in Py-only conditions in comparison to Py/Fu with more than 10× fold-change. Sequence annotation and analysis revealed that many proteins encoded in this cluster are homologous to enzymes involved in dissimilatory sulfite reduction (DSR), as in *Desulfovibrio* sp. (Pires et al., 2006; Pereira et al., 2011). Whether all the 14 genes belong to the same operon is not clear. Indeed, no clear function was predicted for the first four genes (ACL18316-9). Moreover, ACL18319 was not detected at all in the proteomic analysis. Nevertheless, if only considering the proteins ACL18320-9, it is clear that DSR is induced in Py-only conditions (Supplementary Table 5). One explanation for this observation is likely the effect of the addition of sodium sulfide as a reducing agent (at a final concentration of 1 mM) in the anaerobic medium preparation. It is proposed that sulfide is at least partially oxidized abiotically to a variety of sulfur compounds in the medium (Findlay and Kamyshny, 2017), thus likely producing sulfur or sulfite which can be used by *Desulfitobacterium* spp. as electron acceptor (Utkin et al., 1994; Kim et al., 2012). Although the concentration of sulfite remains probably low in the culture medium, it would be enough to induce the expression of the *dsr* gene cluster and possibly also to account for some respiratory metabolism, or at least for an improved energy conservation during fermentation. In a parallel experiment, 1 mM of sodium sulfide was spiked in a culture of strain DCB-2 actively growing in a medium containing pyruvate (and Titanium(III) citrate as alternative reducing agent), the transcription of selected *dsr* genes was induced with 8–15× fold-change (Figure 2), thus in line with the significant up-regulation observed in the proteomic dataset. The behavior of most proteins from the ACL18316-29 gene cluster in the pairwise comparison across all six growth conditions suggests that DSR metabolism is also strongly triggered during respiration with H_2_ as electron donor and in La/ClOHPA conditions (Supplementary Figure 6B). The induction of alternative energetic pathways such as DSR is possibly correlated with the less favorable growth conditions and lower yields observed for these conditions in comparison to Py/Fu and La/Fu, as illustrated by their presence in cluster 3 (Supplementary Figure 3). Furthermore, an additional culture experiment was performed to assess the contribution of the added sodium sulfide to growth in Py-only conditions. Comparing the growth of Py-only cultures with or without sodium sulfide revealed a clear benefit of the addition of 1 mM of sodium sulfide to strain DCB-2, as the extent of growth was significantly higher as compared to the culture devoid of it (grey and blue lines in Supplementary Figure 7A). This was also reflected by the pyruvate consumption and acetate and lactate production of the two cultures (first two panels in Supplementary Figure 7B). Since sulfide itself is not a substrate for *Desulfitobacterium* (but the product of dissimilatory sulfite reduction), the hypothesis that sulfide was chemically oxidized to sulfite in the medium was corroborated by the observation that the addition of 1 mM of sodium sulfite to a culture growing on pyruvate without sulfide could perfectly compensate for the growth deficit observed (orange line in Supplementary Figure 7A). Again, this was confirmed by the almost complete consumption of pyruvate in the culture amended with sulfite (last panel in Supplementary Figure 7B).

Taken altogether, the proteomic dataset of cells growing on pyruvate as sole source of carbon and energy reveals a rather complex metabolism. The complexity is reflected by the diversity and distribution of up-regulated proteins during partial fermentation in comparison to any combination of electron donor and acceptor, and by the small number of proteins commonly up-regulated across all pairwise comparisons. Nevertheless, this analysis mainly revealed proteins catalyzing reactions involved in the central carbon metabolism and in the energy metabolism.

### 3.3. Versatility of respiratory metabolisms: H_2_ or lactate as electron donors

**Figure 3:**
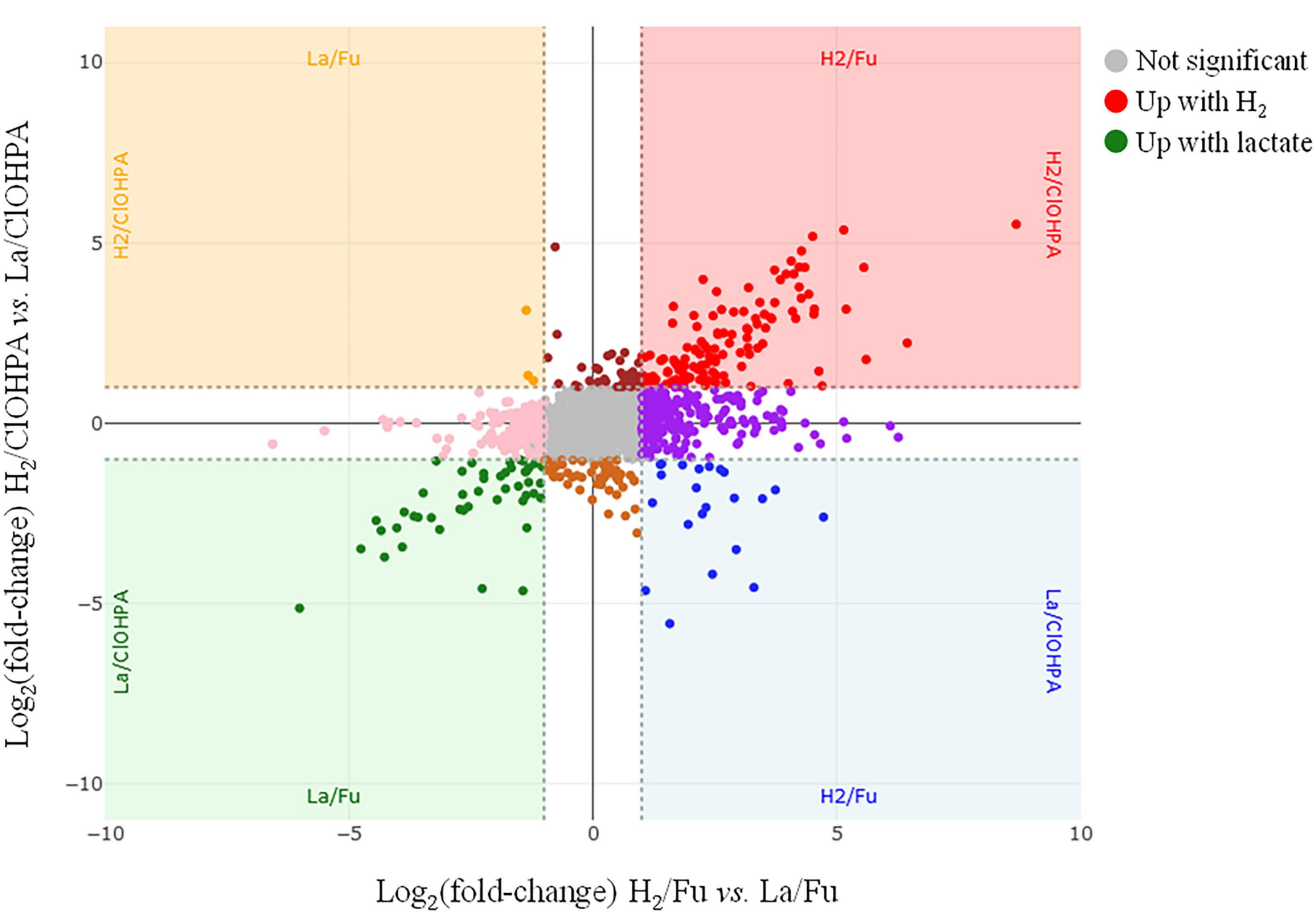
Scatter plot displaying the distribution of the proteins in the pairwise comparison of the proteomes from cells growing on H_2_/Fu vs. La/Fu and H_2_/ClOHPA vs. La/ClOHPA conditions. Note that the proteins significantly up-regulated in H_2_ conditions are displayed in red in the upper right corner, while those induced by lactate are in green in the lower left corner of the scatter plot.

The four growth conditions applied to strain DCB-2 where two electron donors (H_2_ and lactate) and two electron acceptors (fumarate and ClOHPA) were alternatively used allowed us to measure the influence of individual compounds on the proteome. The respective adaptations were highlighted by confronting two pairwise comparisons of the growth conditions representing the proteomic data in scatter plots. For the electron donors, comparing H_2_/ClOHPA vs. La/ClOHPA, and H_2_/Fu vs. La/Fu, a set of 123 proteins was significantly up-regulated and is likely related to H_2_ metabolism, while those that appeared as doubly down-regulated (48 proteins) may play a role in lactate metabolism. The corresponding scatter plot is displayed in Figure 3.

#### 3.3.1. Proteome adaptations to H_2_ as electron donor

The top right panel in Figure 3 displays the 123 proteins that were up-regulated with H_2_ as electron donor in comparison to lactate. The high number of H_2_-specific proteins can be partially explained by the fact that many of them are encoded by genes that are likely part of large operons (Supplementary Table 6). Among them, a selection of proteins is given in Table 2.

**Table 2:** Selected proteins and operons significantly up-regulated with H_2_ as electron donor and acetate as carbon source.

Accession	Predicted Function	H_2_/Fu vs. La/Fu (fold-change)	H_2_/ClOHPA vs. La/ClOHPA (fold-change)
ACL18146	Methylenetetrahydrofolate reductase	3	2
ACL18217	Formate-tetrahydrofolate ligase	4	3
ACL18534	Acetate-CoA ligase	6	9
ACL18581	Fe-containing alcohol dehydrogenase	409	46
ACL18622	Formate-tetrahydrofolate ligase	26	2
ACL18623	Formiminotransferase-cyclodeaminase	16	2
ACL18624	Methylenetetrahydrofolate dehydrogenase (NADP^+^)	17	1,8
ACL18786	Dihydropteroate synthase DHPS	6	13
ACL18944	4Fe-4S ferredoxin iron–sulfur binding domain protein	6	2
ACL18945	Carbon-monoxide dehydrogenase, catalytic subunit	5	2
ACL20816	Dihydropteroate synthase DHPS	6	4
ACL20817	CO dehydrogenase/acetyl-CoA synthase, δ-subunit	5	4
ACL20819	Ferredoxin (cobalt reductive activator)	3	3
ACL20820	CO dehydrogenase/acetyl-CoA synthase, δ-subunit	5	4
ACL20821	CO dehydrogenase/acetyl-CoA synthase, β-subunit	6	4
ACL20822	Carbon-monoxide dehydrogenase, catalytic subunit	4	4
ACL20879	Enoyl-CoA hydratase/isomerase	20	20
ACL20880	Acetyl-CoA acetyltransferase	17	18
ACL20881	Acetyl-CoA hydrolase/transferase	19	27
ACL20883	Protein of unknown function DUF224	4	3
ACL20884	Electron transfer flavoprotein, α-subunit	23	36
ACL20885	Electron transfer flavoprotein, β-subunit	19	11
ACL20886	Acyl-CoA dehydrogenase domain protein	47	20
ACL20887	β-hydroxybutyryl-CoA dehydrogenase	13	19
ACL20888	Acyl-CoA dehydrogenase domain protein	2	4
ACL20889	PAS modulated sigma54 specific transcriptional regulator	4	6
ACL21561	Phosphate butyryltransferase	7	9
ACL21563	Acetyl-CoA acetyltransferase	21	12
ACL21899	Dihydropteroate synthase DHPS	5	4
ACL22273	Formate dehydrogenase formation protein (FdhE)	3	7
ACL22274	Formate dehydrogenase, gamma subunit	4	3
ACL22275	4Fe-4S ferredoxin iron–sulfur binding domain protein	6	3
ACL22276	Formate dehydrogenase, alpha subunit	5	3
ACL22530	Acetyl-CoA hydrolase/transferase	6	6
ACL22531	Acyl-CoA dehydrogenase domain protein	9	9
ACL22532	Acetyl-CoA acetyltransferase	6	8
ACL22533	β-hydroxyacyl-CoA dehydrogenase (NAD-binding)	11	10
ACL22535	Electron transfer flavoprotein, α-subunit	23	9
ACL22536	Electron transfer flavoprotein, β-subunit	10	4
ACL22537	NADH:flavin oxidoreductase/NADH oxidase	3	2

Beforehand, it is noteworthy to recall that cultivating strain DCB-2 with H_2_ as electron donor requires the addition of acetate as carbon source. Indeed, *Desulfitobacterium* spp. are not autotroph, although they can assimilate part of their carbon from CO_2_ (van de Pas et al., 2001). Consequently, the growth of strain DCB-2 with H_2_ and acetate appears as an important driving force in shaping the proteome. Indeed, most of the 123 up-regulated proteins account for approximately half of the proteins present in cluster 4 (Supplementary Figure 3). The following subsections present three major pathways or metabolic functions highlighted under H_2_/acetate: the Wood-Ljungdahl pathway (WLP) for carbon assimilation, two electron-bifurcating pathways likely involved in butyrate metabolism and the unresolved mosaic use of hydrogenases.

##### 3.3.1.1. The Wood-Ljungdahl pathway as preferred route for acetyl-CoA production

**Figure 4:**
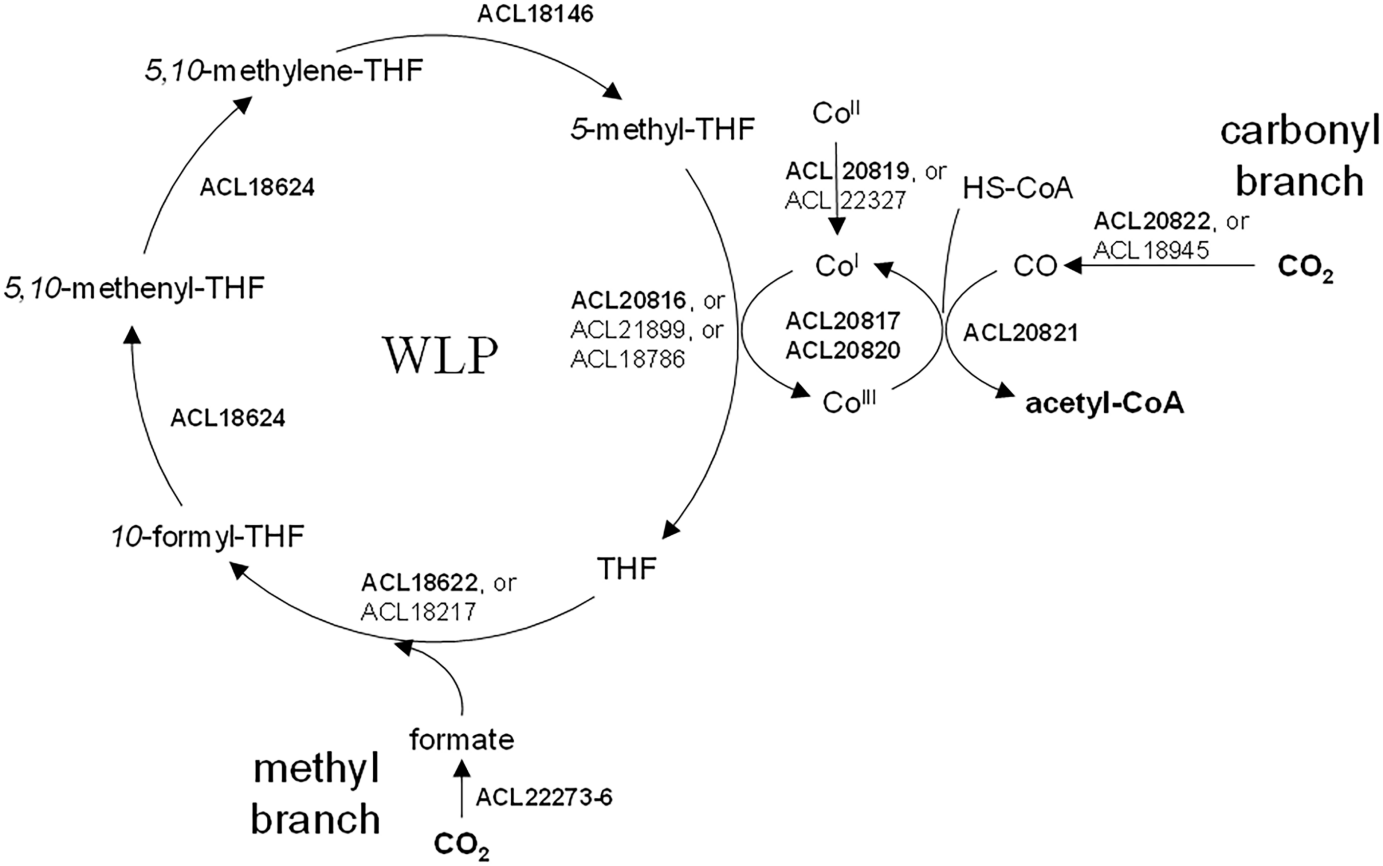
The Wood-Ljungdahl pathway in *Desulfitobacterium hafniense* strain DCB-2. The proteins involved in WLP were identified by sequence homology with the well-characterized enzymes from *Carboxydothermus hydrogenoformans* (Can et al., 2014; Jeoung et al., 2014). All indicated proteins were detected as significantly up-regulated in presence of H_2_ in the medium (as compared to lactate). When several homologs are indicated, the most likely enzyme involved in WLP is indicated in bold. See Table 2 for details on the level of up-regulation.

Many proteins displayed in Table 2 were either directly or indirectly linked to the WLP (Figure 4). Indeed, 6 out of 8 proteins encoded by the CO dehydrogenase/acetyl-CoA synthase (CODH/ACS) operon (ACL20816-22) were significantly up-regulated in H_2_ conditions. Additionally, the three subunits composing the formate dehydrogenase (ACL22274-76) and its associated maturation factor (ACL22273) were also identified as up-regulated, supporting the WLP as previously proposed by Kim et al. (2012)). Also, one or more protein homologues for each step of the methyl branch of the WLP were identified in the H_2_-adapted proteome. Noteworthy is the predicted five-gene operon (ACL18620-4), from which ACL18622 (formate-THF ligase) and ACL18624 (methylene-THF dehydrogenase) participate in the methyl branch of the WLP. The higher expression level of numerous proteins directly or indirectly linked with the WLP in the H_2_-conditions may be a consequence of the co-occurrence of H_2_ and CO_2_ in the medium which could trigger the expression of the pathway. This could also suggest that the bacteria rely on the WLP to produce acetyl-CoA from CO_2_ even though acetate was added as organic carbon source. The first step in the assimilation of acetate, catalyzed by the acetate kinase (ACL21842), is an energy-consuming process and may not be the preferred route in bacteria growing on H_2_ and harboring the WLP. The up-regulation of ACL21842 in lactate conditions compared to H_2_ may corroborate this hypothesis (Supplementary Table 7). In contrast, a predicted acetate-CoA ligase (ACL18534), that could be involved in the active carbon fixation from acetate as proposed earlier (Kim et al., 2012), was systematically up-regulated in H_2_ conditions. Thus, carbon assimilation from acetate is expected to happen in addition to autotrophic CO_2_ fixation through the WLP. Dedicated experiments should be performed to determine the exact nature of the carbon source in strain DCB-2 growing on H_2_. A mixotrophic growth (combination of autotrophic and heterotrophic carbon assimilation mechanisms) has been proposed for *Dehalobacter restrictus* (Holliger et al., 1998) and may also be the case for strain DCB-2.

##### 3.3.1.2. Potential involvement of butyrate metabolism in substrate-level phosphorylation

Two large sets of proteins (ACL20879-89 and ACL22530-38) and a small one (ACL21561-3) contain at least one protein that can be mapped to the butyrate metabolism as described in the KEGG database (map 00650). Both large clusters include predicted flavin-based electron-bifurcating enzymes, a pathway discovered in butyrate-forming anaerobes (Buckel and Thauer, 2018). Moreover, the gene cluster ACL20879-89, and to a lesser extent the ACL22530-38 cluster, show a similar genetic organization as the typical electron-bifurcating gene cluster of *Clostridium kluyveri* that was shown to be involved in butyrate metabolism (Boynton et al., 1996; Seedorf et al., 2008; Supplementary Figures 8A,B). This strongly suggested that strain DCB-2 is able to produce butyrate from acetyl-CoA. Strain DCB-2 is not known as a butyrate-producing organism. However, as a large amount of acetyl-CoA might result from the use of the WLP, it is a possibility that strain DCB-2 uses the electron-bifurcating butyrate synthesis pathway as an additional route for substrate-level phosphorylation. All the proteins possibly involved and that were detected as significantly up-regulated in the H_2_-adapted proteome were mapped to the predicted butyrate pathway (Supplementary Figure 8A). Similarly, in previous proteomic studies on *Desulfitobacterium* spp., electron-transferring flavoproteins (Etf), the basis for electron bifurcation, have been identified (Prat et al., 2011; Kruse et al., 2015). The two Etf subunits homologous to ACL20884 and ACL20885 in *Desulfitobacterium hafniense* strain TCE1 were up-regulated with similar fold-change in batch cultures cultivated on H_2_/PCE in comparison to H_2_/Fu, suggesting that growth in OHR conditions recruited an additional source of energy (Prat et al., 2011). This, however, does not seem to be the case for strain DCB-2 in the H_2_/ClOHPA vs. H_2_/Fu comparison. Nevertheless, *Desulfitobacterium* spp. appear among the bacteria with the highest number of Etf systems, with *Desulfitobacterium hafniense* strain DCB-2 harboring seven of them. Based on the reported classification of Etf proteins, both significantly up-regulated Etf α-and β-subunits (ACL20884-5 and ACL22535-6) belong to the least characterized group 5 of Etf, for which no representative has been yet functionally characterized (Garcia Costas et al., 2017). This reflects the importance of electron bifurcation in *Desulfitobacterium* spp. and the need for further investigation.

##### 3.3.1.3. Numerous hydrogenases detected without any significant regulation pattern

**Figure 5:**
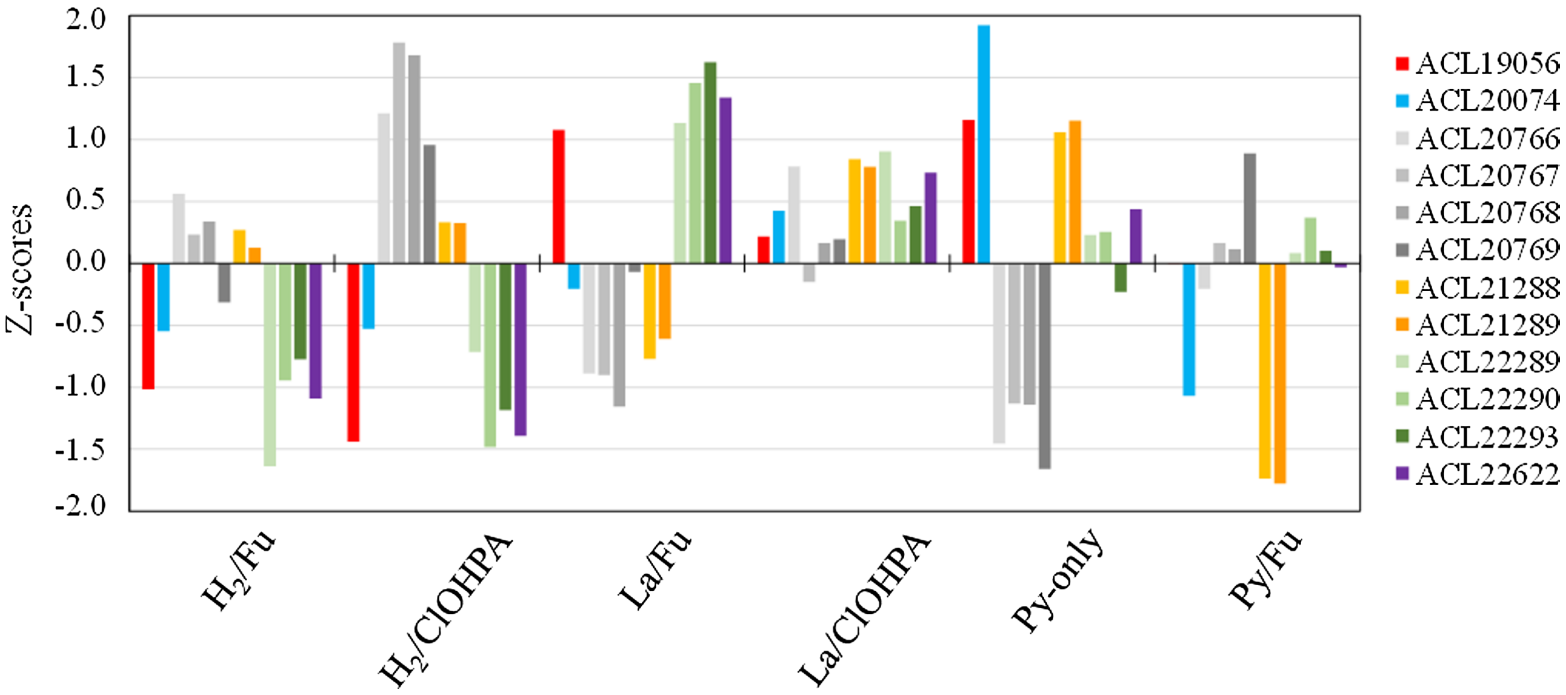
Z-scores of the hydrogenases from *Desulfitobacterium hafniense* strain DCB-2 detected across the six growth conditions. Twelve subunits from six different hydrogenases (out of nine) were detected in the proteomic dataset. The subunits belonging to the same hydrogenase are displayed in different shades of the same color.

Among the nine hydrogenases identified in the genome of strain DCB-2 and classified using the HydDB database (Søndergaard et al., 2016; Supplementary Table 8), none of them was systematically detected as up-regulated in H_2_ conditions when compared to lactate, suggesting that the hydrogen metabolism is needed in all tested metabolisms. This observation is reflected by the wide distribution of the subunits from the six detected hydrogenases in the clustering analysis (Supplementary Table 3). Nevertheless, to better understand the use of these hydrogenases by strain DCB-2, they were plotted according to their relative abundance (expressed as Z-score) in the six growth conditions (Figure 5). It appears that a NiFe hydrogenase (ACL20766-9) seems to be more expressed in H_2_/ClOHPA conditions and is likely to play the role of uptake hydrogenase feeding electrons to the respiratory chain. Although the same is not fully observed in H_2_/Fu conditions, this hydrogenase is clearly down-regulated during the fermentation of pyruvate. The four protein subunits of this hydrogenase correspond to the classical three-subunit membrane-bound complex (HydABC) of NiFe group 1a hydrogenases (Søndergaard et al., 2016) and to an associated maturation factor (HydD). A similar membrane-bound complex has already been identified in the context of OHR in *Desulfitobacterium* (Kruse et al., 2015). By contrast, the relative abundance of the multi-subunits NiFe-hydrogenase belonging to the family of energy-converting hydrogenases (Ech) (ACL22288-93) of group 4f (Søndergaard et al., 2016) appeared less abundant in the two conditions involving H_2_ as electron donor in comparison to lactate. In acetoclastic methanogens, the Ech-type hydrogenase was proposed to produce H_2_ in the cytoplasm at the expense of reduced ferredoxin, while transferring protons through the membrane and contributing to the proton-motive force (Welte and Deppenmeier, 2014). This activity could be induced in strain DCB-2 by the oxidation of lactate to pyruvate and then to acetyl-CoA, however, the source of reduced ferredoxin is unclear.

Additional proteins showing a high up-regulation level due to H_2_ and acetate (Figure 3) are listed in Supplementary Table 6. However, no clear function could be assigned to these proteins (see additional information in the Supplementary material; section 1.1.2).

Taken altogether, the H_2_-specific proteome adaptation is composed mostly by proteins involved in the carbon metabolism rather than in the energy metabolism. This suggests that many observed changes in protein expression is a consequence of the use of acetate as carbon source as opposed to lactate rather than due to the use of H_2_ as electron donor. Nevertheless, the homogenous distribution of H_2_/acetate up-regulated proteins across the six growth conditions into cluster 4 invites to further identify key proteins in this restricted energy metabolism.

#### 3.3.2. Proteome adaptations to lactate as electron donor

In comparison to H_2_, the use of lactate, which can undergo two rounds of oxidation (to pyruvate and then to acetyl-CoA), is probably a significant physiological advantage as it provides energy and carbon at the same time and is readily available to the cells, since it does not require to cross the gas–liquid interface. This might explain the generally better growth performances of strain DCB-2 in lactate conditions (Supplementary Figure 1). In the following subsections are discussed two major proteins directly involved in lactate metabolism: a lactate transporter and a new type of lactate dehydrogenase.

##### 3.3.2.1. Identification of the L-lactate uptake transporter protein

The bottom left panel of the scatter plot in Figure 3 displays the proteins significantly up-regulated when lactate was used as electron donor in comparison to H_2_ in the two pairwise comparisons. A total of 48 proteins were identified there, some of them being encoded by operons (ACL19560-5; ACL22022-9; ACL22619-22) (Supplementary Table 7). This selection of proteins is split between clusters 5 and 9 of the hierarchical analysis (Supplementary Figure 3). The proteins belonging to cluster 9 are grouped together in the top branch of the cluster and are distinct from those in cluster 5 by lower Z-scores in the Py-only proteome. Most remarkably in this protein selection, ACL21424, that occupies the most extreme position in the scatter plot (64× and 35× fold-change up-regulation, respectively), was identified as a possible L-lactate transporter, as already indicated above (section 3.2). In presence of lactate in the medium, we proposed that ACL21424 acts as the major L-lactate uptake transporter. Yet another predicted L-lactate transporter (ACL21426), located in the direct vicinity of ACL21424 and sharing 75% sequence identity with it, was also identified in the proteomic analysis, however it presented a different pattern across the growth conditions. In contrast to the former, ACL21426 was rather abundant in H_2_/Fu condition, suggesting a possible different function. A detailed analysis of the DNA sequence of this region revealed the presence of an *IS4*-type insertion sequence separating the two genes coding for ACL21424 and ACL21426 (Supplementary Figure 9A). In addition, two GntR-type regulators (ACL21422-3), displaying 60% of sequence identity to each other, are encoded directly downstream of ACL21424 and are homologous to *Bacillus subtilis* LutR protein, that has been shown to regulate the lactate utilization operon (Chai et al., 2009). While both L-lactate transporters share a high level of sequence identity, their promoter regions are different (Supplementary Figure 9B), in line with their alternative use by strain DCB-2.

##### 3.3.2.2. Involvement of an unusual lactate dehydrogenase complex

A classical *lutABC* operon is present in the genome of strain DCB-2 (ACL21278-80), and resembles that of *Campylobacter jejuni* (Taylor and Kelly, 2019), *Bacillus subtilis* (Chai et al., 2009) and *Shewanella oneidensis* (Pinchuk et al., 2009), all of them being involved in L-lactate oxidation to pyruvate (Supplementary Figure 9C). Although these proteins were detected in the proteome of strain DCB-2, they did not show any particular pattern in any of the conditions tested. In contrast, two proteins (ACL21106-7) with yet unknown function appeared in cluster 5 with relatively high fold-changes in both pairwise comparisons when lactate was present (Supplementary Figure 9C). Sequence analysis revealed that ACL21107 is homologous to LutC, while ACL21106 appears as a fusion of LutB and LutA, here designated as Lut[BA] (Supplementary Figure 9C). According to Taylor et al., the Lut proteins could serve as L-lactate oxidase receiving electrons from lactate at the cytoplasmic face of the membrane, transferring them *via* FeS clusters present in LutB and *via* the conserved CCG motifs in LutA, and reducing menaquinones in the membrane (Taylor and Kelly, 2019). Redox potential considerations suggested that this type of lactate oxidase is electroneutral (with H^+^/e^−^ = 0; Taylor and Kelly, 2019), thus not contributing to the establishment of a proton gradient. This latter function is likely fulfilled by the subsequent oxidation of pyruvate to acetyl-CoA feeding electrons to the complex I-like enzyme (M. Willemin & J. Maillard, unpublished data). Nevertheless, we propose that ACL21106-7 form the major lactate dehydrogenase (LDH) involved in the electron-transport chain of strain DCB-2 growing on lactate.

A striking feature is that the majority of the proteins in the lactate-specific corner of the scatter plot (Figure 3) were also significantly up-regulated in Py/Fu or Py-only conditions when compared to H_2_ conditions (Supplementary Table 7). This is likely due to the direct physiological link between lactate and pyruvate in the metabolism. However, the direct comparison of the proteomes obtained in La/Fu and Py/Fu conditions helped confirming the key role of the identified lactate transporter (ACL21424) and the Lut[BA]C complex (ACL21106-7) in lactate metabolism (Supplementary Table 9). Interestingly, the DSR pathway that was highlighted in cells growing in less favorable growth conditions (Supplementary Figure 6), was also significantly up-regulated in La/Fu vs. Py/Fu. This tends to confirm that lactate oxidation to pyruvate requires an additional energy kick, as already discussed above.

To conclude with the pairwise comparisons of lactate- vs. H_2_-specific proteomes, differences were mainly highlighted in the carbon and/or central metabolism and in the direct uptake machinery. It shows the importance of alternative energy conservation pathways in cells growing in H_2_ conditions with acetate as carbon source. Beside the identification of unusual proteins, such as the Lut[BA]C complex for lactate oxidation, no clear evidence was obtained for substrate-specific electron-transfer moieties in the respiratory chain. This agrees with the existing OHR model, in case of the use of hydrogen as electron donor, proposing that the uptake hydrogenase transfers electrons directly to the menaquinone pool which transfers them to the final reductase. In contrast, when lactate is used, it is not clear whether an electron shuttle is needed to transfer electrons from the predicted cytoplasmic Lut[BA]C complex to the menaquinone pool in the membrane.

### 3.4. Versatility of respiratory metabolisms: Fumarate and ClOHPA as electron acceptors

**Figure 6:**
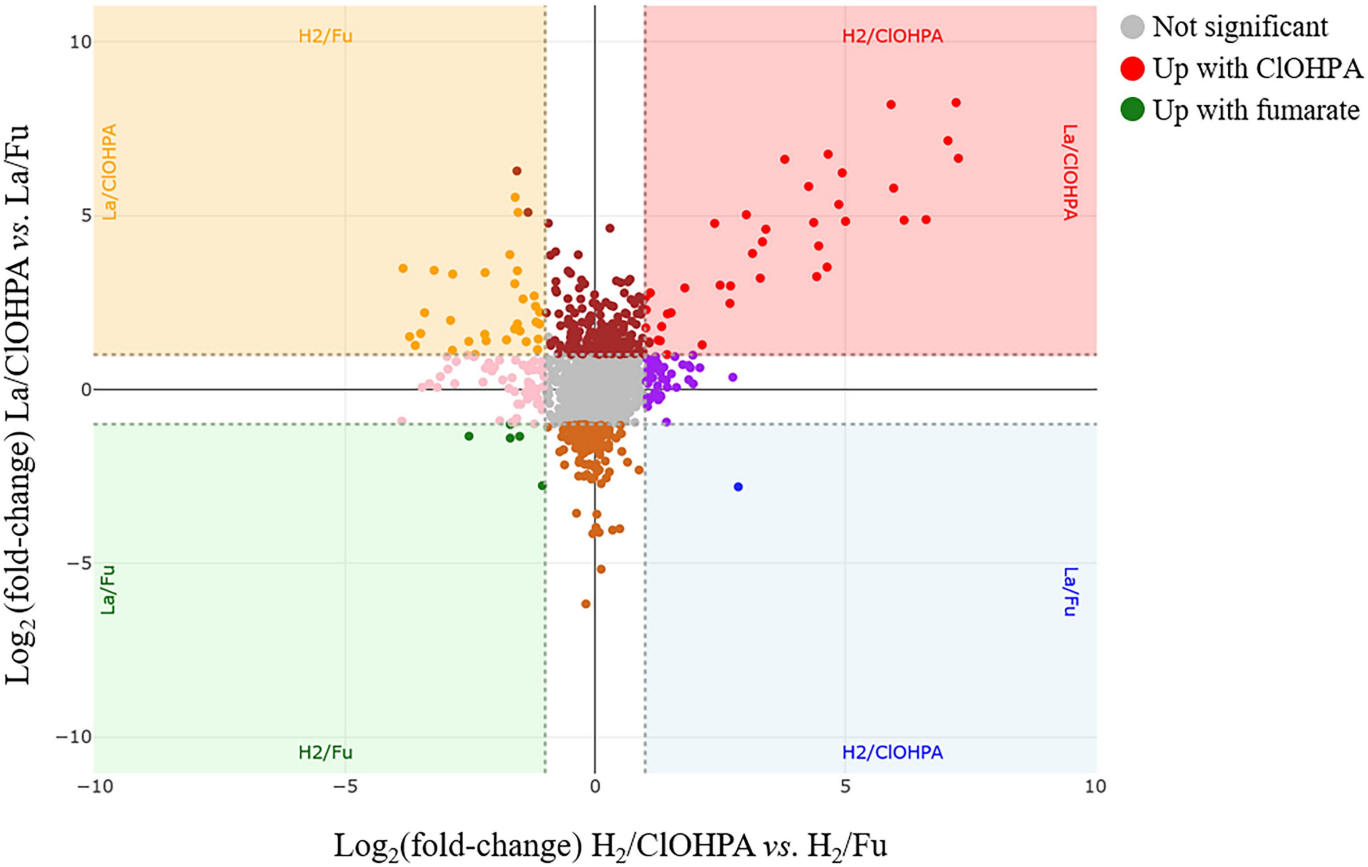
Scatter plot displaying the distribution of the proteins in the pairwise comparison of the proteomes from cells growing on H_2_/ClOHPA vs. H_2_/Fu and La/ClOHPA vs. La/Fu conditions. Note that the proteins significantly up-regulated in ClOHPA conditions are displayed in red in the upper right corner, while those induced by fumarate are in green in the lower left corner of the scatter plot.

Another couple of pairwise comparisons of the proteomes were investigated to highlight the adaptations of strain DCB-2 to the electron acceptor, either fumarate or the organohalide ClOHPA. The scatter plot displaying the relative abundance of proteins in La/Fu vs. La/ClOHPA and H_2_/Fu vs. H_2_/ClOHPA conditions is depicted in Figure 6. The proteome adaptations are presented in separated subsections relative to the electron acceptor. In line with the observation made in the pyruvate-only conditions, it is important to note that the DSR pathway may also contribute to the energy conservation in the respiratory metabolisms with hydrogen as electron donor and in OHR in general (Supplementary Table 3.

#### 3.4.1. Proteome adaptations to fumarate as electron acceptor

Only very few proteins were identified as significantly up-regulated in the fumarate quarter of the scatter plot (depicted on the bottom left corner in Figure 6 and listed in Supplementary Table 10). None of them displayed a fold-change ratio above 5 and no unambiguous function can be attributed to them. Moreover, when expanding the analysis across other pairwise comparisons, the relative abundance of these proteins did not appear systematically up-regulated in the fumarate condition.

##### 3.4.1.1. The quinol:fumarate reductase as part of a diverse family of oxidoreductases in strain DCB-2

From the fumarate-specific proteome, one could expect to identify the dissimilatory fumarate reductase (FRD, also known as quinol:fumarate reductase, QFR), the terminal redox enzyme involved in fumarate respiration. QFR protein complexes belong to the large family of succinate:quinone oxidoreductases (SQOR) and have been characterized for a few bacteria [for a review, see (Lancaster, 2013)]. Surprisingly, in our dataset while 22 of the 30 members of the SQOR family present in the genome of strain DCB-2 could be quantified, none of them was identified as up-regulated in the pairwise comparisons. ACL18807-9 appear as the closest homologues to the characterized membrane-bound FrdABC proteins from *Wolinella succinogenes* (Kröger et al., 2002; Kruse et al., 2015), as shows the analysis of the diversity of SQOR flavoproteins identified in strain DCB-2 (Supplementary Figure 10). ACL18808 is the catalytic flavoprotein, ACL18807, the *b*-type cytochrome and ACL18809 is the FeS protein. However, the pattern of ACL18808-9 (ACL18807 was never detected) across the growth conditions indicates a slightly higher relative abundance due to the presence of H_2_ than that of fumarate (Supplementary Table 6). A peculiar pattern has been already reported for the homologous FRD proteins in *Desulfitobacterium dehalogenans* (Desde_0617–8), as they were up-regulated in formate/ClOHPA and formate/fumarate growth conditions when compared to Py-only, but not in formate/fumarate vs. formate/ClOHPA (Kruse et al., 2015). Nevertheless, the possible involvement of ACL18807-9 in anaplerotic reactions linked to the reductive TCA cycle, as proposed earlier (Kim et al., 2012), may explain a constitutive production of the QFR enzyme.

#### 3.4.2. Proteome adaptations to OHR with ClOHPA as electron acceptor

Contrasting with the small number of proteins found up-regulated in the fumarate conditions, 40 proteins appeared significantly up-regulated in the top right corner of the scatter plot in Figure 6 (i.e., in presence of ClOHPA, when opposed to fumarate). Half of them consist of proteins that are part of or directly adjacent to reductive dehalogenase (*rdh*) gene clusters (Supplementary Table 11; Supplementary Figure 11A). A stricter evaluation of the proteomic adaptation to ClOHPA was also considered when both growth conditions involving ClOHPA as electron acceptor were compared to all four non-ClOHPA conditions (Supplementary Table 12; Supplementary Figure 11B). From 40 proteins, the number of overall up-regulated proteins dropped to 25, among which 19 belong to *rdh* gene clusters. Most of these proteins fall into a single branch of cluster 3 in the hierarchical analysis (Supplementary Figure 3), highlighting the homogeneous behavior of OHR-related proteins.

##### 3.4.2.1. The *rdh-5* and *rdh-6* gene clusters as key respiratory pathways for ClOHPA

Proteins from four out of the seven different *rdh* gene clusters were identified in this restricted selection. Most of the proteins encoded in the *rdh* gene the rdh-5 gene clusters (ACL18776-83) and 6 (ACL18796-804) were detected and quantified. Noteworthy, and similarly to previous studies (for a review, see (Türkowsky et al., 2018)), is the systematic lack of the RdhB subunits in the proteomic dataset that correspond to the putative membrane anchor of the cognate key catalytic enzyme, RdhA. As expected, proteins from cluster 6, known to be dedicated to the respiration of ClOHPA (Smidt et al., 2000; Gábor et al., 2006), showed the highest significant up-regulation factors with RdhA6 (ACL18801) being more expressed in H_2_/ClOHPA with up to 818× fold-change in comparison to La/Fu (Supplementary Table 11). There, the CRP/FNR-type regulatory protein RdhK6 (ACL18797) is expected to induce the transcription of the *rdh* gene cluster 6, as shown earlier (Gábor et al., 2006, 2008). Surprisingly, all the detected proteins of cluster 5 also displayed high expression ratios in conditions fed with ClOHPA compared to non-ClOHPA conditions, highlighting a possible cross-talk of RdhK6 with the *rdh* gene cluster 5 or the induction of this cluster by the RdhK5 regulator that responds to the presence of ClOHPA. Possible cross-talk across *rdh* gene clusters have been postulated earlier for strain DCB-2 (Gábor et al., 2008; Willemin et al., 2020).

##### 3.4.2.2. New corrinoid-related proteins induced by OHR metabolism?

**Figure 7:**
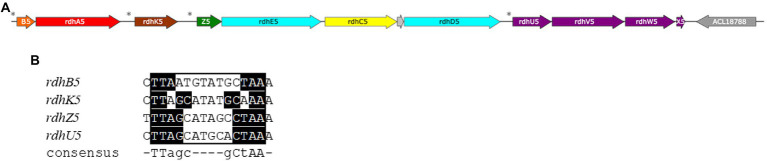
The reductive dehalogenase (*rdh*) gene cluster 5 from *Desulfitobacterium hafniense* strain DCB-2 (loci ACL18776-87). **(A)** Map of the gene cluster consisting of four putative transcriptional units: *rdhBA*, *rdhK*, *rdhZECD* and *rdhUVWX*. **(B)** Identification of partially conserved DNA motifs, so-called dehaloboxes, Gábor et al. (2006) upstream of each transcriptional unit, as indicated by the stars in panel A, that are targets of the transcriptional activator protein, RdhK5. (Note: a short additional locus was predicted between *rdhC5* and *rdhD5* (ACL18782, grey arrow), without any evidence that it codes for a protein.)

More interestingly, four proteins encoded by genes that are directly downstream of the *rdh*-5 gene cluster (ACL18784-7; Figure 7A) appeared with similar up-regulation factors as the latter. None of the proteins encoded in the loci ACL18784-7 shows homology to any protein already belonging to OHR metabolism (Kruse et al., 2016). Therefore, we propose to assign new names to the corresponding proteins as RdhU, RdhV, RdhW and RdhX, respectively. The results of protein sequence analysis are presented in Table 3. RdhU shows approximately 42% of sequence identity with members of a corrinoid protein family involved in methylamine metabolism in *Methanosarcina* spp. (Krätzer et al., 2009). While RdhV has no match in UniProt, it displays similarity with the uroporphyrinogen decarboxylase (UroD), an enzyme likely involved in the maturation of the corrin ring. RdhW (ACL18786) shows 32% of sequence identity with the MeTr enzyme from *Moorella thermoacetica* that catalyses the methyl transfer from methyltetrahydrofolate to the corrinoid cofactor of a corrinoid/FeS protein, possibly RdhU here. Finally, ACL18787 codes for a short 48-aa long protein (RdhX) that does not display any sequence similarity in UniProt. One additional feature that links the *rdhUVWX* gene cluster to OHR metabolism is the identification of the partially conserved palindromic DNA sequence in the promoter region of *rdhU* that is likely the target of the RdhK5 regulator (Figure 7B). In other OHRB, a similar gene cluster (*rdhUVX*, however lacking *rdhW*), was identified in the genome of *Dehalobacter restrictus* strain PER-K23 in three copies. While the first copy (with loci AHF10407-9) is located directly in-between the *rdh* gene clusters 13 and 14, the other two copies (AHF10446-8 and AHF10449-51) are the result of gene duplication and are located directly upstream of the *rdh* gene cluster 23. Several of the corresponding proteins have been identified by proteomics in *Dehalobacter restrictus* growing with PCE as electron donor (Rupakula et al., 2013), further highlighting their involvement in OHR metabolism. Whether these proteins are participating to the biosynthesis, the homeostasis, the assembly of the corrinoid cofactor into reductive dehalogenases or any other function remains to be elucidated.

**Table 3:** Sequence analysis of newly assigned Rdh proteins.

Protein name	Accession	Best match in UniProt (ID)	Description	References
RdhU	ACL18784	Q8TTB0.1 (42%)	methylamine corrinoid protein	Krätzer et al. (2009)
RdhV	ACL18785	–	Uroporphyrinogen decarboxylase (UroD)	
RdhW	ACL18786	Q46389.2 (33%)	Methyltransferase (MeTr)	
RdhX	ACL18787	–	Unknown function	

##### 3.4.2.3. Further cross-talk among *rdh* gene clusters

Among Rdh proteins, ClOHPA also seems to induce the production of the reductive dehalogenase RdhA4 (ACL18775), a chemotaxis sensory transducer (RdhO1, ACL18750), and the transcriptional regulators RdhK1 (ACL18751) and RdhK7 (ACL20644). Earlier work in *rdh* gene regulation has already shown that 3-chloro-4-hydroxybenzoate, a compound highly similar to ClOHPA, induces the transcription of *rdhA4* and *rdhA5* genes in addition to *rdhA6* (Kim et al., 2012). Moreover, 2,4-dichlorophenol (2,4-DCP) that has been added to the culture of strain DCB-2, has also been reported as inducer of *rdhA4*, *rdhA5* and *rdhA6* genes (Mac Nelly et al., 2014). The *ortho* position of the chlorine atom that is released from 2,4-DCP and the *para*-position of the second substituent (a chlorine atom in 2,4-DCP and the acetic acid group in ClOHPA) may explain the similar induction observed with 2,4-DCP as with ClOHPA. This induction pattern observed with *para*-substituted *o*-chlorophenols across several *rdh* gene clusters indicates a certain degree of cross-talk in the OHR metabolism of strain DCB-2, a hypothesis that we proposed earlier (Maillard and Willemin, 2019). In contrast to 2,4-DCP, 2,6-dichlorophenol (2,6-DCP) harbors two chlorines in *ortho*-position and appears to selectively induce the *rdh-5* gene cluster in strain DCB-2 (Mac Nelly et al., 2014). It is important to note here that the cross-talk across *rdh* gene clusters may be the result of two possible mechanisms: the recognition of a single organohalide compound by several RdhK transcriptional regulators (that leads to the induction of their cognate *rdh* gene cluster) and the possible recognition of several DNA motifs (so-called dehaloboxes (Mazon et al., 2007)) located in different *rdh* gene clusters by a single RdhK regulator. The knowledge on the OHR regulation network is still in its infancy and requires substantial biochemical investigation. The strategy of using RdhK hybrid proteins for deciphering its specific organohalide and DNA partners represents one way to address this complex question (Willemin et al., 2020).

Beside the Rdh proteins discussed above, six additional proteins were significantly up-regulated by ClOHPA (Supplementary Table 11) and discussed in section 1.1.5 of Supplementary material: ACL18593-4, ACL18768, ACL19741, ACL21973 and ACL21997.

## 4. Conclusion

The genus *Desulfitobacterium* presents the most versatile metabolism among bacteria capable of organohalide respiration. Their genome offers a very large array of redox enzymes potentially involved in energy metabolism. With the present comparative proteomic study, we aimed at identifying the key proteins of *Desulfitobacterium hafniense* strain DCB-2 responsible for some of its typical energy metabolic pathways. Our proteomic dataset is characterized by one of the highest resolutions obtained so far with OHR bacteria and revealed several new metabolic features of strain DCB-2 across six different growth conditions.

The first surprise was the induction of the dissimilatory sulfite reduction (DSR) in energetically rather unfavorable conditions. A major outcome of the present study is that strictly fermentative growth with pyruvate may only be achieved if no sulfide is used. It appears indeed that the addition of sodium sulfide as reducing agent in the strictly anaerobic medium contributes significantly to growth most probably as a result of abiotic oxidation of sulfide to sulfite, which can be used as terminal electron acceptor in a respiratory metabolism or at least as electron sink, thus cycling sulfite back to sulfide. Therefore, one should consider using an alternative reducing agent with *Desulfitobacterium* spp. if fermentative conditions are exclusively investigated in the future.

Several metabolic pathways were significantly triggered in the proteomes of *Desulfitobacterium hafniense* strain DCB-2 when it grew on the different combinations of electron donors and acceptors. The Wood-Ljungdahl pathway appears as key player in carbon assimilation in the presence of H_2_, despite the fact that acetate was added (and is required) as carbon source. It reinforces the observation that *Desulfitobacterium* spp. as well as many other OHRB display a mixotrophic regime. Regarding the lactate metabolism, a transporter was clearly identified for the uptake of L-lactate (ACL21424), while the lactate dehydrogenase (LDH) highlighted in the proteomic dataset (ACL21106-7, Lut[BA]C) may represents a new type of LDH where two of the typical subunits are fused together. Fumarate respiration did not induce any particular redox enzyme. Thus, it is not clear whether ACL18807-9, as a member of the largely diversified succinate:quinone oxidoreductases (SQOR), acts as the membrane-bound fumarate reductase in strain DCB-2. In contrast, another member of the SQOR family (ACL21973) was significantly induced under organohalide respiration, confirming previous proteomic analyses, however not allowing us to assign a clear function. The most relevant output of the comparative proteomics regarding OHR metabolism is the identification of four new proteins (ACL18784-7, designated as RdhU, -V, -W and-X) likely involved in the biosynthesis and assembly of the corrinoid cofactor into RdhA enzymes.

Altogether, we believe that our proteomic dataset represents a new landmark in the understanding of metabolic adaptations of *Desulfitobacterium* spp. in particular, as well as that of facultative organohalide-respiring bacteria in general.

## Data availability statement

The datasets presented in this study can be found in online repositories. The names of the repository/repositories and accession number(s) can be found in the article and in Supplementary material.

## Author contributions

MW: conducted the experiments, analyzed the data, and wrote the manuscript. RH: conducted the proteomic analyses. FA: analyzed the proteomic dataset. CH: conceived the study and revised the manuscript. JM: conceived the study, conducted the experiments, analyzed the data, and wrote the manuscript.

## Funding

The research was funded by the Swiss National Science Foundation (SNSF) in the frame of the SNF Project No. 31003A_173059.

## Conflict of interest

The authors declare that the research was conducted in the absence of any commercial or financial relationships that could be construed as a potential conflict of interest.

## Publisher’s note

All claims expressed in this article are solely those of the authors and do not necessarily represent those of their affiliated organizations, or those of the publisher, the editors and the reviewers. Any product that may be evaluated in this article, or claim that may be made by its manufacturer, is not guaranteed or endorsed by the publisher.

## Footnotes

^1^ www.ebi.ac.uk/pride/
