# Supplementary material for: Proteome adaptations of the organohalide-respiring Desulfitobacterium hafniense strain DCB-2 to various energy metabolisms
Source: Front Microbiol. 2023 Jan 17;14:1058127. doi: 10.3389/fmicb.2023.1058127 (PMC9888536; doi:10.3389/fmicb.2023.1058127)
Supplement: Supplementary file 2 [file Presentation_1.PDF]

Supplementary Material

1 Supplementary Data

1.1 Additional proteomic data interpretation

Beside the specific proteins that are discussed in the paper, more information and interpretation can be found for the various energy metabolisms considered in this study.

1.1.1 Partial fermentation of pyruvate

Proteins from five successive genes (ACL21108-12) were significantly up-regulated in Py-only with high level (between 5× and 30× fold-change, depending on the protein and conditions). On the one hand, ACL21108, which is likely encoded by a monocistronic gene, is annotated as a NAD-dependent decarboxylating malate dehydrogenase (dMDH, E.C. 1.1.1.38). In contrast to the classical malate dehydrogenase (E.C. 1.1.1.37) that produces oxaloacetate from malate in the TCA cycle, dMDH is usually involved in anaplerotic pathways and produces pyruvate from malate, as reported earlier (1). While this activity is not likely to be required in presence of pyruvate in the medium, ACL21108 may function in the reverse direction and produces malate from pyruvate, CO_2 and NADH, thus replenishing TCA cycle intermediates. On the other hand, ACL21109-12 correspond to a predicted operon (in reverse orientation) coding for proteins likely involved in the metabolism of citrate and succinate. ACL21110 and ACL21112 show 40% and 35% sequence identity with the small (α) and large (β) subunits, respectively, of the succinyl-CoA synthetase (SucC and SucD, SCS) from *E. coli*. SCS catalyses the conversion of succinyl-CoA to succinate in the TCA cycle. The lack of the α-ketoglutarate dehydrogenase in strain DCB-2 was reported earlier (1), thus interrupting the TCA cycle, requires an alternative source of succinyl-CoA for anabolism. While no function could be assigned to ACL21111, ACL21109 displays some sequence homology to the β subunit of the citryl-CoA lyase (CitE) from *E. coli* or the malonyl-CoA thioesterase from *Rhodobacter sphaeroides*. While the former catalyses together with CitD and CitF the citrate lyase activity in *E. coli* that is involved in citrate fermentation (2,3), the latter has been proposed to participate in the assimilation of acetyl-CoA via the ethylmalonyl-CoA pathway and glyoxylate (4). The exact substrates of ACL21109 and ACL21110/2 cannot be inferred from sequence homology. However, taken together, these five proteins are likely to play a role in anaplerotic reactions, in accordance to the hypothesis that strain DCB-2 runs the TCA cycle in a reductive biosynthetic direction (1).

Among the 19 proteins generally up-regulated in Py-only condition, there are also two uncharacterized transcriptional regulators (ACL19672, ACL22391) and three proteins encoded by a series of three consecutive genes (ACL22517-9). Although no clear function could be predicted, the sequence of ACL22518-9 indicate that they are redox enzymes, suggesting a possible involvement in the energy metabolism of strain DCB-2 cells growing with pyruvate only.

A lactate transporter (ACL21424) was highly up-regulated in the pairwise comparison of Py-only with Py/Fu, but also when Py-only is compared to H_2/Fu or H_2/ClOHPA. On the contrary, it was not up-regulated when compared to lactate conditions. This suggests that ACL21424 is involved in both lactate uptake and secretion. The uptake function may occur when lactate is used as electron donor, while lactate needs to be secreted as a fermentation product of pyruvate.

Figure 1 also shows subsets of 25 and 14 proteins that are up-regulated in Py-only conditions in comparison to all other conditions except La/ClOHPA or H2/Fu, respectively. While the latter subset mainly consists of proteins harbouring radical SAM domain and other cofactors likely involved in anabolism. Here two clusters of proteins are noteworthy: a six-gene cluster (ACL21175-80) encoding a member of the complex iron-sulfur molybdoenzyme (CISM) superfamily and two subunits of a putative NiFe hydrogenase five-gene cluster (ACL21187-91). Representatives of the CISM superfamily in bacteria are extremely diverse in function and harbour various gene composition (5). Interestingly, a genomic survey has identified *Desulfitobacterium hafniense* to have the largest set of molybdoenzymes, most of them belonging to the diverse DMSO reductase subfamily (6). A thorough sequence analysis suggests that the ACL21175-80 gene cluster forms an operon, which displays, beside the typical CISM subunits A (the catalytic molybdoenzyme) and B (a ferredoxin), another copy of the B subunit and an enzyme showing low sequence homology to the NAD(P)H-nitrite reductase (NirB) or the CoA/CoA-disulfide reductase (CoADR). Therefore, this composite cluster of redox enzymes may indicate that, beside Py-only conditions, strain DCB-2 exploits additional sources of energy from minor components of the medium. The NiFe hydrogenase, that is encoded in ACL21287-91, contains, in addition to the typical large subunit and Tat-dependent small subunit, a Tat-dependent *c*-type cytochrome (ACL21287), and two proteins with unknown function. The replacement of *b*-type cytochrome by a *c*-type cytochrome in NiFe H_2-uptake hydrogenases has been reported for most δ-Proteobacteria (7). The *c*-type cytochrome, which was also found up-regulated in fermentation (however only when compared to Py/Fu and La/Fu conditions), belongs to the family of split-Soret diheme *c*-type cytochromes (Ssc) (8) and may well receive electrons from the associated NiFe hydrogenase as it was proposed in the metabolism of *Desulfovibrio desulfuricans* (9). The fact that the up-regulation level of this hydrogenase is substantially higher when comparing to Py/Fu and La/Fu conditions than to H_2 conditions may reflect the common need of hydrogenases in fermentative metabolism (likely producing H_2) and in respiration with H_2 as electron donor. Proteome adaptations to La/ClOHPA in comparison to all other conditions indicate that it shares some metabolic pathways with fermentation, suggesting that cells growing with lactate and ClOHPA display a mixed energy metabolism with both respiratory and fermentative aspects. It is likely that ClOHPA is reduced from the oxidation of lactate to pyruvate via respiration, but also possible that the resulting pyruvate is then fermented.

Supplementary Table 4 also reports on several proteins that are highly up-regulated in Py-only conditions but do not display a clear function. Noteworthy, however, are the proteins ACL20816, -17 and -20, which belong the gene cluster ACL20816-23 that is mainly responsible for the Methyl

branch of the Wood-Ljungdahl pathway (WLP). These proteins are clearly up-regulated in Py-only conditions, but also in respiration with H_2 as electron donor.

The catalytic flavoprotein subunit (ACL18746, ACL20535 and ACL21007) of three members of the succinate:quinone oxidoreductase family (SQOR, (10)) were also systematically up-regulated in Py-only conditions (Table 1). These three flavoproteins (out of 31 homologs encoded in the genome of strain DCB-2) display diverse genetic environments. None of them display the classical 4- or 3-gene operons as for the succinate dehydrogenase (SdhABCD) and fumarate reductase (FrdABC), respectively, but all three of them harbour a Twin-arginine translocation (Tat) signal peptide and a lipoprotein signal peptide, indicating that these proteins are exported across and anchored at the cytoplasmic membrane. ACL18746 and ACL21007 appear as monocistronic genes, while ACL20535 displays a possible membrane-bound partner (ACL20534). So far, neither a function nor a specific substrate can be associated with any of the three flavoproteins.

1.1.2 H_2 as electron donor

ACL18581, annotated as alcohol dehydrogenase appeared with the most extreme position in the H_2-specific corner of the scatter plot (Figure 3), as it displays 409× and 46× fold-changes in up-regulation in H_2/Fu *vs*. La/Fu and in H_2/ClOHPA *vs*. La/ClOHPA conditions, respectively. This protein shows 57% sequence identity with the second alcohol dehydrogenase (ADH II) of *Zymomonas mobilis*, which is likely involved in acetaldehyde reduction to ethanol (11) and responsible for intracellular NAD^+/NADH homeostasis (12). The addition of acetate in the growth medium of strain DCB-2 growing with H_2 as electron donor is likely responsible for the up-regulation of ACL18581. Indeed, acetate directly or acetyl-CoA as an intermediate of the WLP (see section 3.3.1.1 in the paper) could induce the production of the alcohol dehydrogenase, and thus helps restoring the NAD^+ pool in the cell.

ACL18082, a protein potentially involved in the vitamin B6 synthesis, appeared with a high level of up-regulation (between 35- and 41-fold more expressed in H_2-conditions). The over-expression of vitamin biosynthesis might indicate a poor energetic state of the cells, which maybe try to compensate for important missing intermediates. This same reason might explain the presence of two other proteins (ACL19102-03) which seem to be involved in the active transport of solutes. Arguments to explain the presence of the latter examples in the H_2-specific selection still need to be further investigated.

Additionally, the ferredoxin (ACL21793), also present in the H_2-specific adaptation, could represent an important alternative electron shuttle in the H_2-related energy metabolism.

In addition, an alternative way to produce *5,10*-methenyl-THF is provided by ACL19451, which was also up-regulated. Interestingly, the two proteins directly following ACL19451, initially annotated as NuoE (ACL19452) and NuoF (ACL19453), were also up-regulated with a similar behaviour. Although not up-regulated in H_2/ClOHPA vs La/ClOHPA, ACL19454, a molybdoenzyme of the CISM family, is likely part of the same operon (ACL19451-4). ACL19452-4 is likely to form an NAD^+-dependent formate dehydrogenase, similarly to the FdsABG complex identified in

Rhodobacter capsulatus (13). This suggests that the two proteins ACL19452-3 could be indirectly associated with the WLP rather than participating to the complex I-like enzyme, as previously proposed (14). Indeed, the genetic organisation and proteomic dataset here suggest that these two proteins are not co-occurring and not interacting with the 11-subunit complex I-like enzyme identified in *Desulfitobacterium hafniense* (M.S. Willemin, PhD thesis, EPFL).

A four-genes cluster (ACL22329-32) appear to be relatively highly up-regulated. As an example, ACL22330 is found 32-times more expressed in H_2/ClOHPA compared to La/ClOHPA. Interestingly, this cluster was previously identified as a putative *O*-demethylase operon (15). The methyltransferase subunit (ACL22331) encoded by this operon is homologous to MtgB in *Desulfitobacterium hafniense* strain Y51, which has been shown to use glycine betaine as methyl donor to methylate tetrahydrofolate (THF), which can be oxidised to CO_2 and serve as alternative electron donor in respiration (16). The involvement of an *O*-demethylase in the use of phenyl methyl ethers such as vanillate to follow the methyl branch of WLP in the reverse direction was also proposed for strains DCB-2 and Y51 in the context of growth on vanillate (1). Alternatively, this route represents also a way to by-pass the methyl branch of the WLP by producing methyl-THF which can enter the carbonyl branch directly leading to the production of acetate. The growth medium applied here, however, does not contain any source of glycine betaine. Therefore, the physiological role of this gene cluster in the adaptation of the proteome toward the use of H_2 remains to be elucidated.

1.1.3 Lactate as electron donor

Besides being up-regulated in lactate *vs*. H_2 condition, the proteins ACL21106-7 also appeared as up-regulated in lactate conditions *vs*. Py/Fu but not *vs*. Py-only (Supplementary Table 6). The fact that the LDH would be present in fermentative conditions at similar level as when lactate is the electron donor can be explained by the production of lactate from pyruvate in the lactic acid fermentation pathway, which could induce the expression of the LDH. The same situation was observed also for the lactate transporter (ACL21424), which indicates that in the Py/Fu conditions, all the pyruvate is converted to acetyl-CoA and thus there is no need for lactate-specific enzymes as no lactate is produced.

To reveal the true lactate-dependent proteome, the separate volcano plots were considered that compared La/Fu vs. Py/Fu and La/Fu *vs*. Py-only conditions. Most of the proteins up-regulated in the lactate *vs*. H_2 pairwise comparisons (Supplementary Table 6) appeared at the centre of the volcano plots while only one protein (ACL22622) was specifically up-regulated by the presence of lactate. The up-regulated proteins identified in the pairwise comparisons of La/Fu *vs*. Py/Fu and La/Fu *vs*. Py-only conditions (Supplementary Table 7) revealed, beside ACL22622, only two additional common proteins, namely ACL18111 and ACL21412. The former is a periplasmic solute binding protein, possibly involved in lactate uptake, while the latter is a DAHP synthase involved in the shikimate pathway of aromatic amino acids biosynthesis.

The eight proteins of the ACL22022-9 predicted operon involved in the uridine monophosphate synthesis pathway (*pyr* operon) from glutamine showed an average 8× fold-change significant up-regulation in lactate conditions compared to H_2 conditions (Supplementary Table 7). Overall, the *pyr* operon organisation is similar to that of *B. subtilis* (Supplementary Figure 12A), the regulation of which has been studied (17). RNA structure prediction of the 5'-untranslated region of *pyrR* (ACL22030) strongly suggests that, as in *B. subtilis*, transcriptional attenuation of the *pyr* operon occurs in strain DCB-2 when pyrimidines are in excess through the action of the regulatory PyrR protein (Supplementary Figure 12B). Furthermore, this pathway was systematically up-regulated in any condition compared to H_2 conditions, suggesting a stronger need of pyrimidine nucleotides in conditions where strain DCB-2 grows faster.

Finally, the six proteins encoded by the ACL19560-5 loci were identified as significantly up-regulated (~3× fold-change in average) in the lactate-specific corner of the scatter plot (Figure 3). These proteins likely form a membrane-bound protein complex displaying the typical A, B and C subunits of a complex iron-sulfur molybdoenzyme (CISM) together with a dedicated molecular chaperone (ACL19564) and two tandem-domain rhodanese proteins (ACL19560 and ACL19565). The homologous proteins were already described in *Desulfitobacterium hafniense* strain TCE1, where it was proposed to be involved in sulfur metabolism, possibly as polysulfide reductase (18). At the time, only one copy of the rhodanese subunits (corresponding to ACL19565) was detected with a 4× fold-change in La/PCE in comparison to La/Fu (19). In contrast, the present analysis identified all the proteins from this CISM cluster. Up-regulation of these proteins was also observed when pyruvate was used as electron donor (Py/Fu), thus suggesting that this cluster is rather down-regulated in H_2 conditions and during fermentation of pyruvate (Py-only), as illustrated by their position in cluster 9. A member of the CISM family has been proposed to be part of the quinone-independent OHR respiratory chain in *Dehalococcoides mccartyi* (20), which triggered the interest for this type of membrane-bound complexes in the metabolism of OHRB. However, it is still unclear whether ACL19560-5 is part of the electron transfer chain in strain DCB-2 growing with organic electron donors, or whether it represents an alternative reductase with sulfur compounds as terminal electron acceptors.

1.1.4 Fumarate as electron acceptor

It was observed that the data from H_2/Fu condition displayed a strong discrepancy, thus precluding the possibility to identify proteomic adaptations to fumarate. Therefore, another protein selection was considered where the proteomes of all fumarate-containing conditions (with exception of H_2/Fu) were compared to that of non-fumarate conditions (Supplementary Table 11), it resulted in a selection of 23 proteins which mostly belong to cluster 9 of the hierarchical analysis (Supplementary Figure 3). From this analysis, it was clear that eight from the eleven proteins (ACL19523-33) involved in inosine monophosphate (IMP) biosynthesis pathway (as part of the purine metabolism) were significantly up-regulated in presence of fumarate. Although it is not clear why this is induced by fumarate, one could investigate the link between the presence of fumarate (or succinate as the product of the fumarate reductase) and anabolic reactions derived from intermediates of the TCA cycle. Interestingly, ACL22922, which is homologous to ACL19525, was also slightly up-regulated

together with ACL22921. Both proteins are likely involved in the interconversion of IMP to adenine monophosphate (AMP), thus participating to the AMP/fumarate homeostasis in the cell. Two additional clusters of proteins are noteworthy from this selection, namely ACL20506-7 and ACL22378-81. The former is involved in the production of threonine from aspartate (which is derived from fumarate), while the latter proteins are encoded by a 7-gene cluster that is responsible for the conversion of chorismate to tryptophan.

1.1.5 ClOHPA as electron acceptor

An interesting observation from Supplementary Table 11 that is yet difficult to explain, was made from the pairwise comparison of the two growth conditions with ClOHPA as electron acceptor. Although both conditions are triggering OHR metabolism, most of Rdh proteins encoded by the gene clusters 5 and 6 display a clear significant up-regulation when H_2, in comparison to lactate, is used as electron donor. To some extent also, these proteins were up-regulated in other H_2 *vs*. lactate or pyruvate in pairwise comparisons. This suggests that a stricter energy regime tends to induce OHR metabolism as if the bacterium needs to be more prompt to use organohalides when the growth conditions are generally less favourable. This observation may be in line with the metabolism of obligate OHR bacteria such as *Dehalobacter* spp. and *Dehalococcoides* spp. (see (21) for a review on OHR regulation).

Six additional proteins were significantly up-regulated by ClOHPA (Supplementary Table 11): ACL18593-4, ACL18768, ACL19741, ACL21973 and ACL21997. Three of these proteins are related to the stress response. ACL19741 is the small heat shock protein Hsp20, ACL18594 and ACL21997 are peptidases, the former being the ATP-dependent Clp protease proteolytic subunit (ClpP). The up-regulation of these in the presence of ClOHPA seems to indicate that OHR is associated with a certain degree of stress, as already proposed for PCE for *Desulfitobacterium hafniense* strain TCE1 (22). Noteworthy also is ACL21973, a flavoprotein belonging to the large family of succinate:quinone oxidoreductases (SQOR, (23)). This protein is homologous to Desde_3368 from *Desulfitobacterium dehalogenans* that has been detected as clearly up-regulated upon growth with ClOHPA (24). While the gene encoding the flavoprotein is likely transcribed as a monocistronic mRNA, the protein displays a typical lipoprotein signal peptide with the cysteine-containing motif LAGC. It is therefore expected that ACL21973 is exported across the cytoplasmic membrane, where its signal peptide is cleaved off and the protein is anchored by lipidation of the cysteine residue. Sequence analysis using the UniProt database revealed that this protein shows some similarity to the characterized urocanate reductase (UrdA, SO_4620) of *Shewanella oneidensis* (25). UrdA has been shown to harbour two flavin prosthetic groups, one covalently-bound FMN in the N-terminal domain of the protein and one non-covalently bound FAD in the C-terminal domain. Effectively, ACL21973 displays the motif for binding FMN covalently to the conserved threonine residue (DX$_3$GA**T**) (26). No substrate can be assigned to ACL21973 since none of the conserved residues forming the active site of UrdA is conserved. Whether ACL21973 participates to the OHR metabolism remains to be elucidated.

2 Supplementary Figures and Tables

2.1 Supplementary Figures

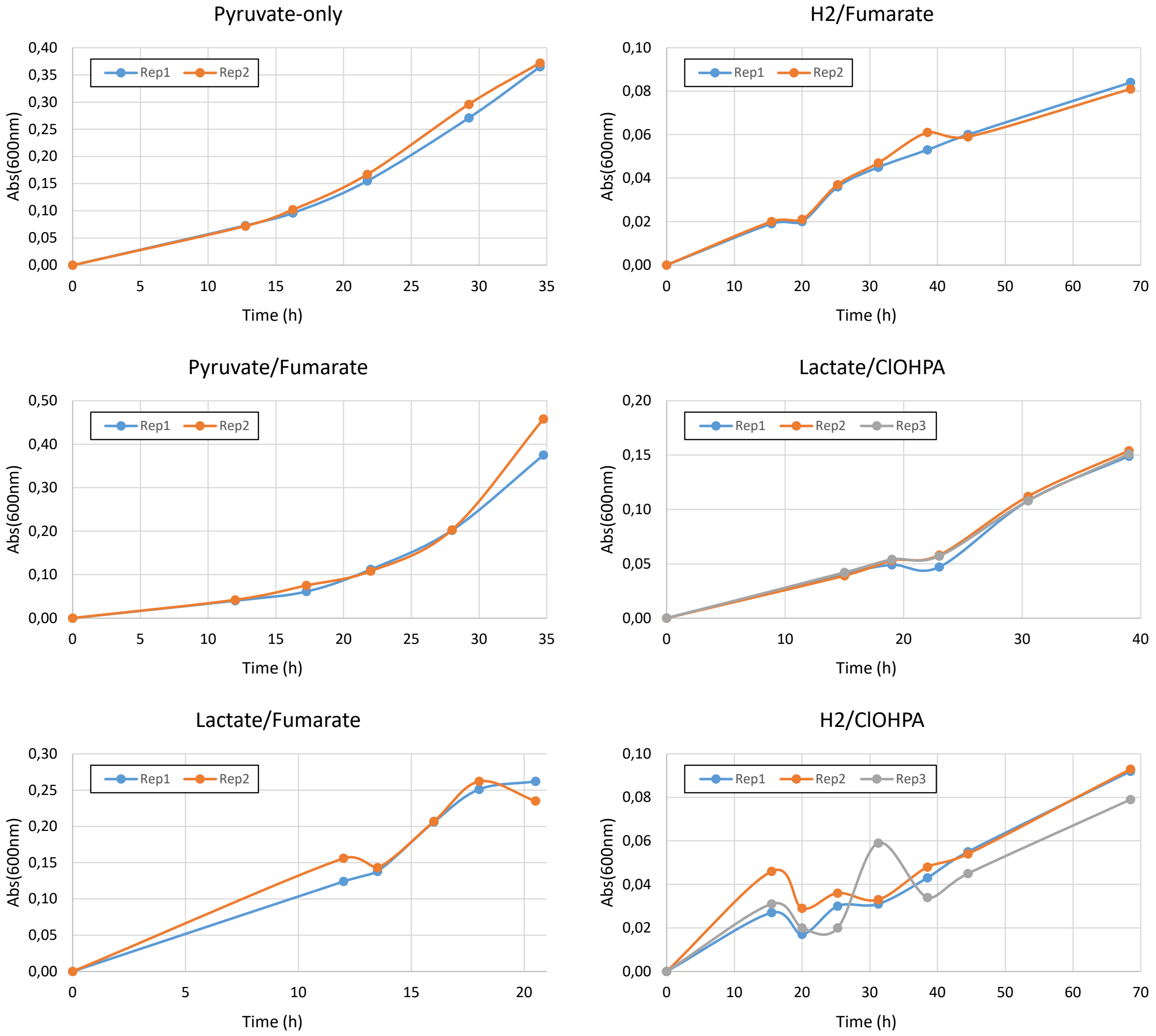

Supplementary Figure 1. Typical growth curve of *Desulfitobacterium hafniense* strain DCB-2 cultivated in various growth conditions. The absorbance at 600 nm was monitored for duplicate or triplicate cultures in each condition.

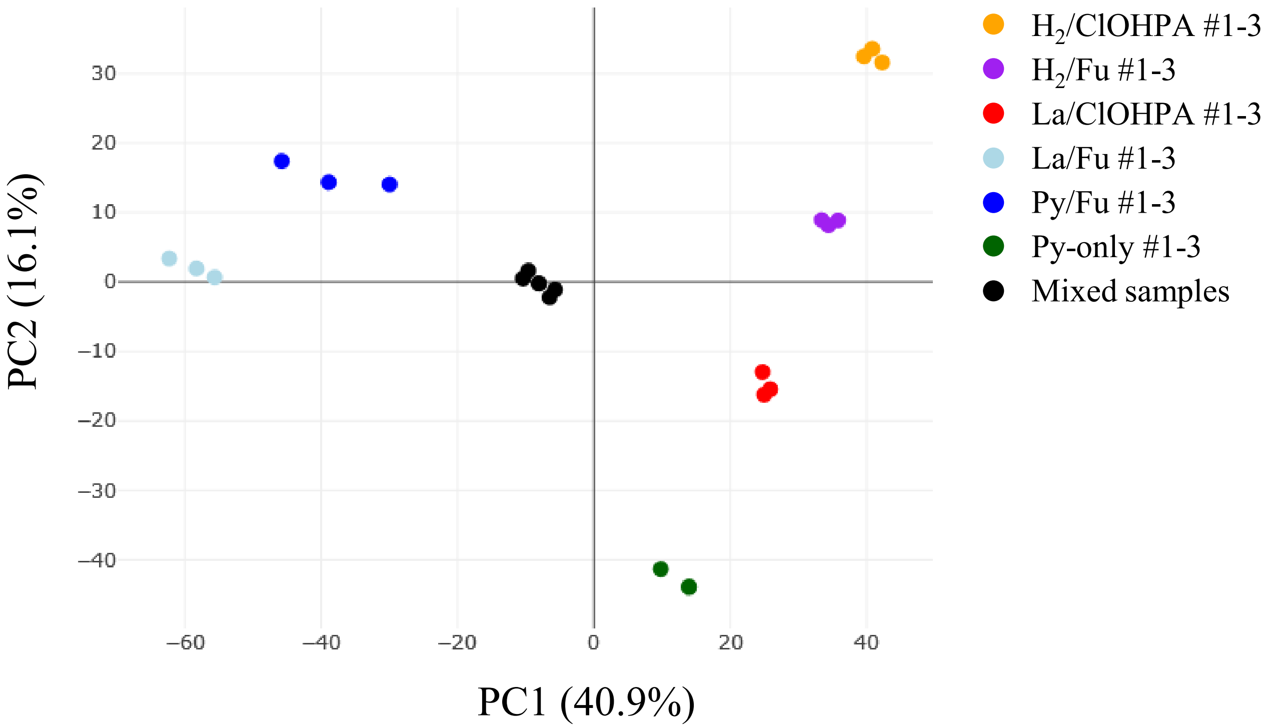

Supplementary Figure 2. Principal component analysis (PCA) of the proteomes issued from *Desulfitobacterium hafniense* strain DCB-2 cells cultivated in six different growth conditions, each of them in triplicates.

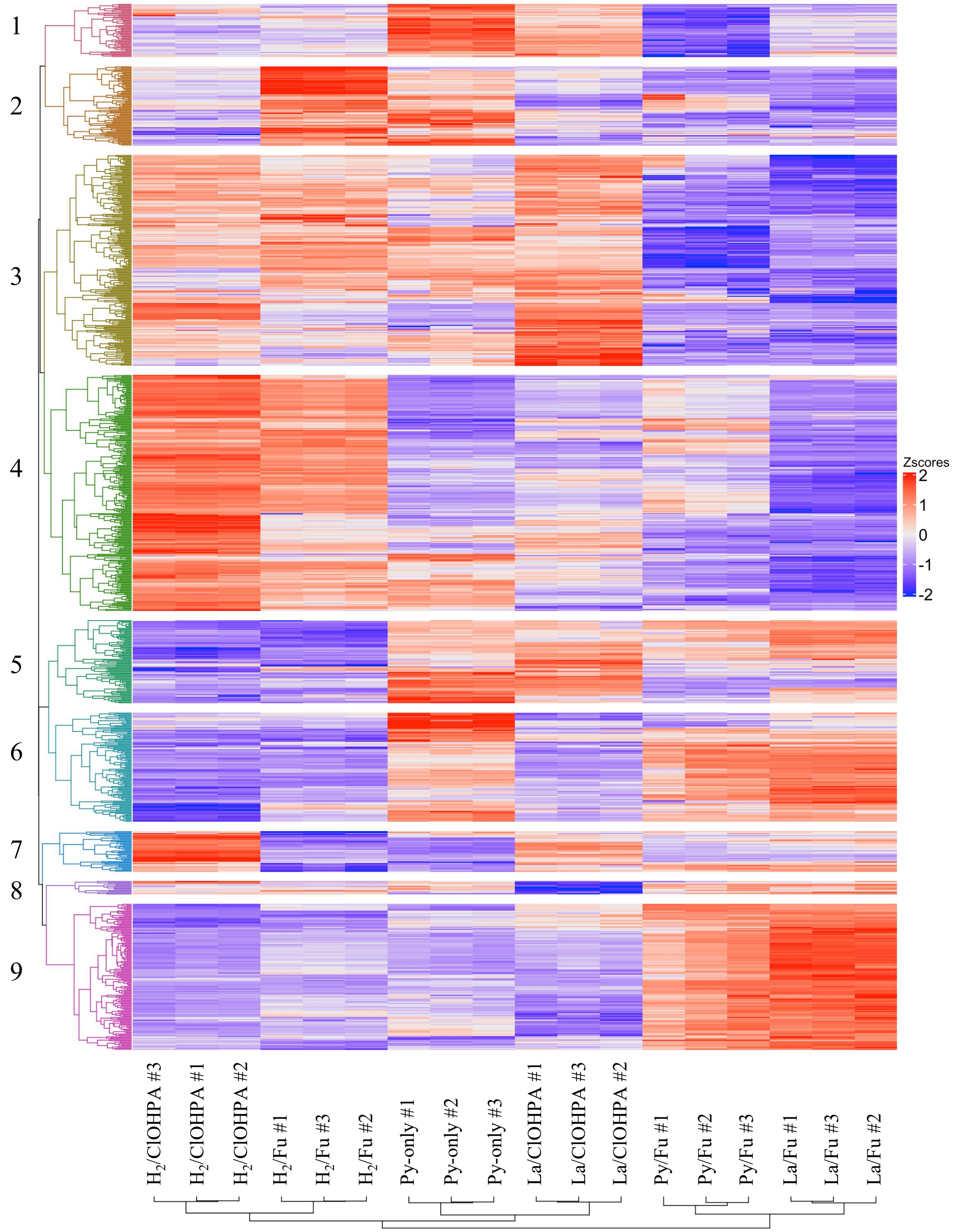

Supplementary Figure 3. Heatmap of the normalised TMT protein abundancies (calculated as *Z*-scores) quantified in the proteomes of *Desulfitobacterium hafniense* strain DCB-2 cells that were cultivated in six different growth conditions (each of them in triplicates). The number of 9 clusters that was chosen as the best representation of the different abundancy patterns.

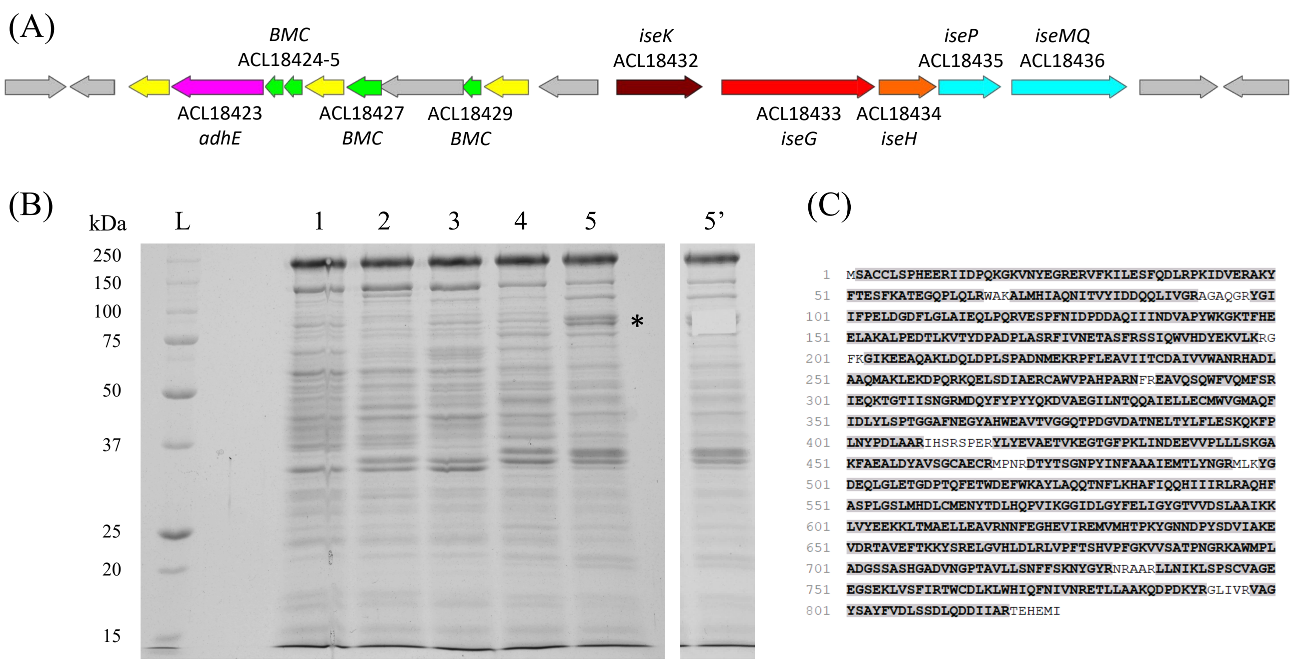

Supplementary Figure 4. *Desulfitobacterium hafniense* strain DCB-2 and isethionate metabolism. (**A**) Genomic neighbourhood of isethionate (*ise*) metabolising genes in *Desulfitobacterium hafniense* strain DCB-2. Genes coloured in yellow are homologous to subunits of the ethanolamine and propanediol utilisation enzymes. Genes coloured in green belong to the family of bacterial microcompartment (BMC) structural genes. The gene in pink is homologous to the acetaldehyde dehydrogenase (*adhE*) of *Bilophila wadsworthia*. (**B**) Protein profiles of cell-free extracts from *Desulfitobacterium hafniense* strain DCB-2 growing in various conditions, as analysed by SDS-PAGE. The star indicates the expected position of ACL18433 (IseG) in sample *5*. (**C**) Peptide coverage of ACL18433 from LC-MS/MS analysis of the excised gel piece. Identified peptides are shaded in grey. Legend: *L*: protein ladder; *lane 1*: pyruvate-only (with Na_2S as reducing agent); *lane 2*: pyruvate-only (no Na_2S); *lane 3*: pyruvate + 1 mM sodium sulfite; *lane 4*: lactate + 5 mM sodium sulfite; *lane 5*: lactate + 10 mM isethionate; *lane 5’*: same as sample 5 showing the gel piece that was cut for performing LC-MS/MS analysis.

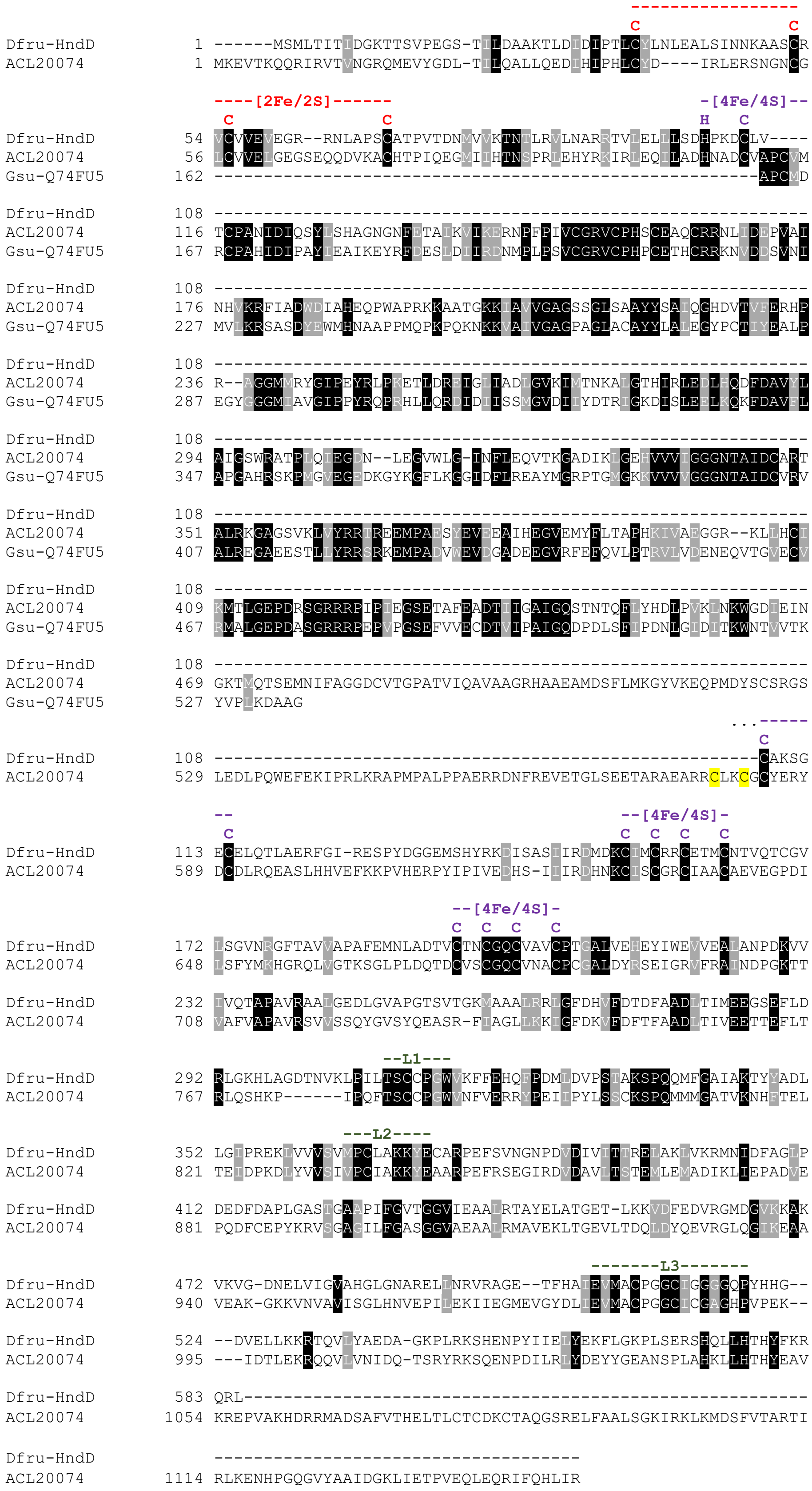

Supplementary Figure 5. Alignment of ACL20074 from *Desulfitobacterium hafniense* strain DCB-2 with HndD from *Desulfovibrio fructosovorans* (*Dfru*-HndD) and with a portion of SfrB from *Geobacter sulfurreducens* (*Gsu*-Q74FU5). Conserved sequence features are indicated above the sequence alignment.

(A)

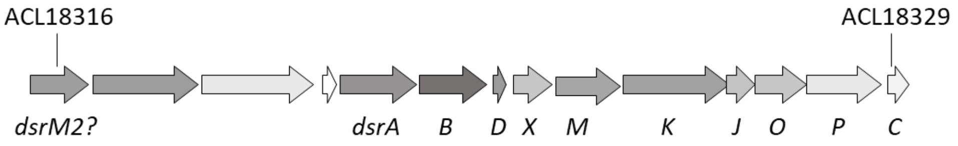

(B)

Accession	Predicted protein function	Py *vs.* Py/Fu	Py *vs.* La/Fu	Py *vs.* H_2/Fu	Py *vs.* H_2/ClOHPA	Py *vs.* La/ClOHPA	La/Fu *vs.* Py/Fu	H_2/Fu *vs.* Py/Fu	H_2/ClOHPA *vs.* Py/Fu	La/ClOHPA *vs.* Py/Fu	H_2/Fu *vs.* La/Fu	H_2/ClOHPA *vs.* La/Fu	La/ClOHPA *vs.* La/Fu	H_2/ClOHPA *vs.* H_2/Fu	H_2/Fu *vs.* La/ClOHPA	H_2/ClOHPA *vs.* La/ClOHPA
ACL18316	Nitrate reductase, γ subunit (DsrM-like)	17	4	1	1	1	4	29	27	17	7	7	4	1	2	2
ACL18317	Protein of unknown function (ferredoxin?)	18	4	1	1	1	4	29	27	16	7	7	4	1	2	2
ACL18318	FAD-dependent NAD-disulphide oxidoreductase	3	2	1	1	1	1	4	5	4	3	3	3	1	1	1
ACL18319	Ferredoxin	nd	nd	nd	nd	nd	nd	nd	nd	nd	nd	nd	nd	nd	nd	nd
ACL18320	Dissimilatory sulfite reductase, α subunit (DsrA)	23	5	1	1	1	5	38	31	18	8	7	4	1	2	2
ACL18321	Dissimilatory sulfite reductase, β subunit (DsrB)	40	6	1	1	1	6	66	55	32	11	9	5	1	2	2
ACL18322	Dissimilatory sulfite reductase, unknown function (DsrD)	19	4	1	1	1	4	20	16	14	5	4	3	1	1	1
ACL18323	Hypothetical protein (DsrX)	8	2	1	1	1	4	11	8	9	3	2	2	1	1	1
ACL18324	Dissimilatory sulfite reductase (DsrM)	13	4	1	1	1	3	19	14	11	7	5	4	1	2	1
ACL18325	Dissimilatory sulfite reductase, 4Fe-4S ferredoxin (DsrK)	15	4	1	1	1	4	23	16	13	6	4	4	1	2	1
ACL18326	Dissimilatory sulfite reductase (DsrJ)	8	5	1	1	1	2	14	10	7	8	6	4	1	2	1
ACL18327	Dissimilatory sulfite reductase, 4Fe-4S ferredoxin (DsrO)	8	4	1	1	1	2	11	8	7	5	4	3	1	2	1
ACL18328	Dissimilatory sulfite reductase (DsrP)	3	2	1	1	1	1	4	3	2	3	2	2	1	2	1
ACL18329	Dissimilatory sulfite reductase, γ subunit (DsrC)	2	1	0	1	1	1	3	3	2	2	2	1	1	2	1

Supplementary Figure 6. The dissimilatory sulfite reduction (*dsr*) gene cluster of *Desulfitobacterium hafniense* strain DCB-2. (**A**) Organization of the *dsr* gene cluster. (**B**) Overall pairwise comparison (fold-change) of the *dsr* encoded proteins as identified in the proteomes of the different growth conditions.

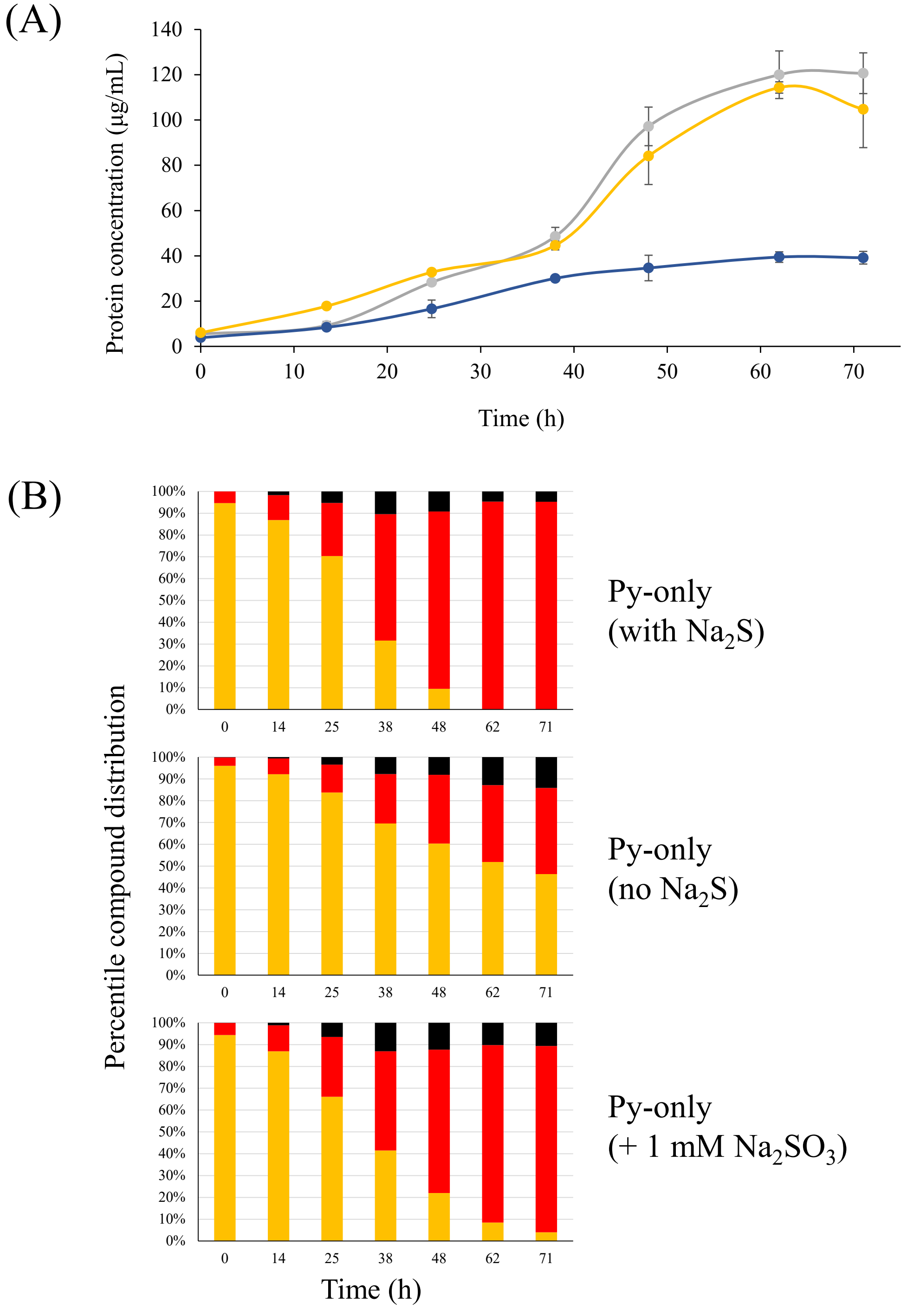

Supplementary Figure 7. Monitoring of *Desulfitobacterium hafniense* strain DCB-2 cultures in various growth conditions. (**A**) Evolution of protein concentration as proxy for growth. Grey line: Py-only (with Na_2S); blue line: Py-only (no Na_2S); orange line: Py-only (+ 1 mM sodium sulfite). (**B**) Evolution of pyruvate (orange bars), acetate (red bars) and lactate (black bars) in the growth medium of the three cultures. Concentration data are expressed as percentile distribution of the various compounds.

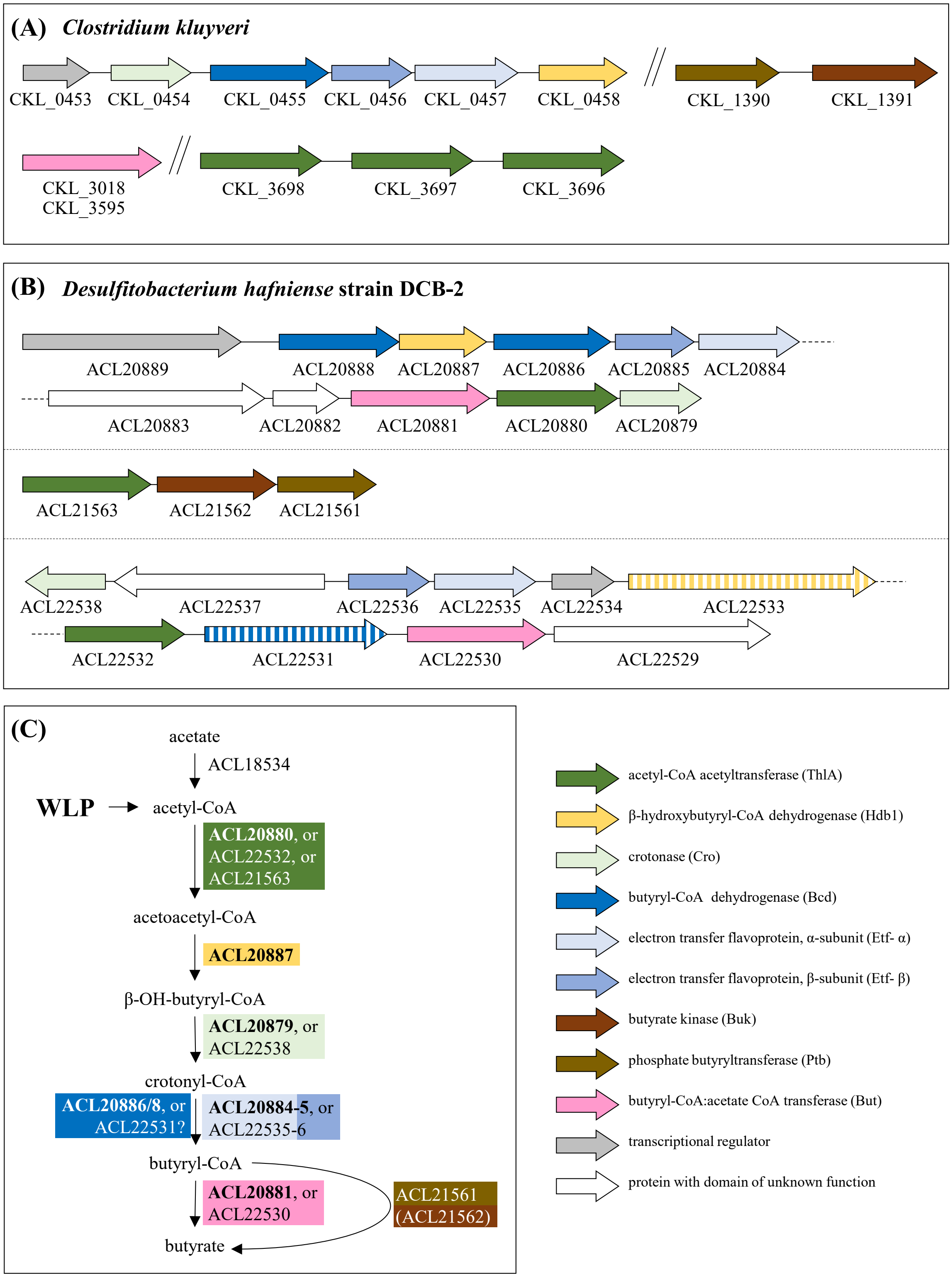

Supplementary Figure 8. Butyrate metabolism. (**A**) Gene clusters involved in butyrate metabolism in the well-characterized *Clostridium kluyveri* (27). (**B**) Gene clusters putatively involved in butyrate metabolism in *Desulfitobacterium hafniense* strain DCB-2. (**C**) Predicted metabolic pathway of butyrate production in *Desulfitobacterium hafniense* strain DCB-2.

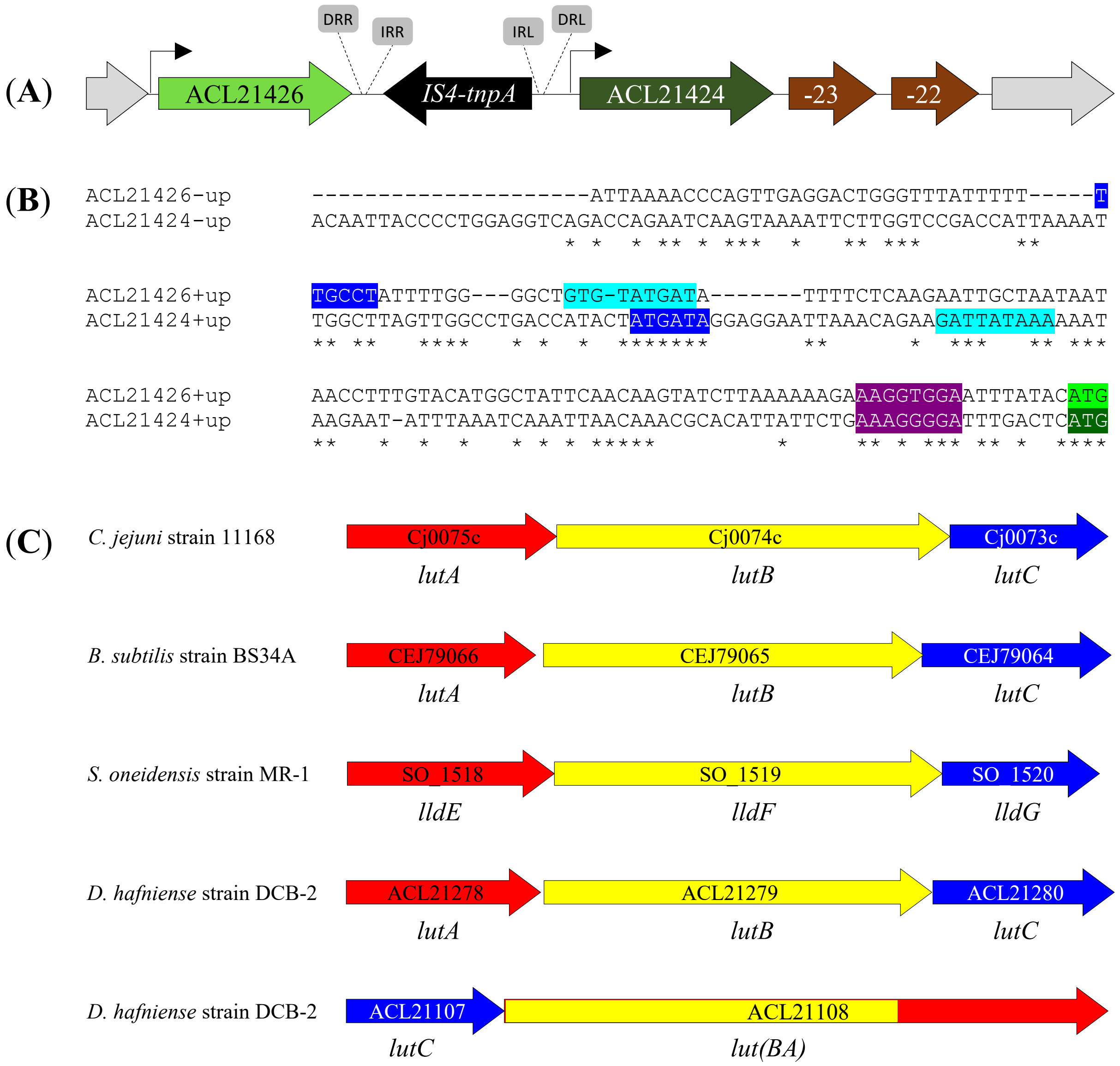

Supplementary Figure 9. The genetic region coding for both L-lactate transporters and lactate-utilisation in *Desulfitobacterium hafniense* strain DCB-2. (**A**) Genetic organization of loci ACL21422-6 showing the two genes encoding L-lactate transporters (in green), a transposase (in black), features of the *IS4*-type insertion sequence (such as the inverted repeats and the direct repeats), and the genes coding for two GntR-type regulators (in brown). (**B**) Sequence alignment of the promoter regions upstream of ACL21426 and ACL21424 loci. Colour code: light and dark green: start codon of both genes; purple: predicted ribosome-binding site; light and dark blue: predicted -10 and -35 regions of the promoters, respectively. (**C**) LutABC encoding genes. *Desulfitobacterium hafniense* strain DCB-2 harbours two operons coding for lactate-utilisation proteins: a classical *lutABC* operon (ACL21278-80), similar to that of *Campylobacter jejuni*, *Bacillus subtilis* and *Shewanella oneidensis*, and an unusual operon made of two genes, *lutC(BA)*, the latter being a fusion of *lutB* and *lutA*. Note that this is the unusual *lut* operon that was strongly up-regulated in lactate conditions.

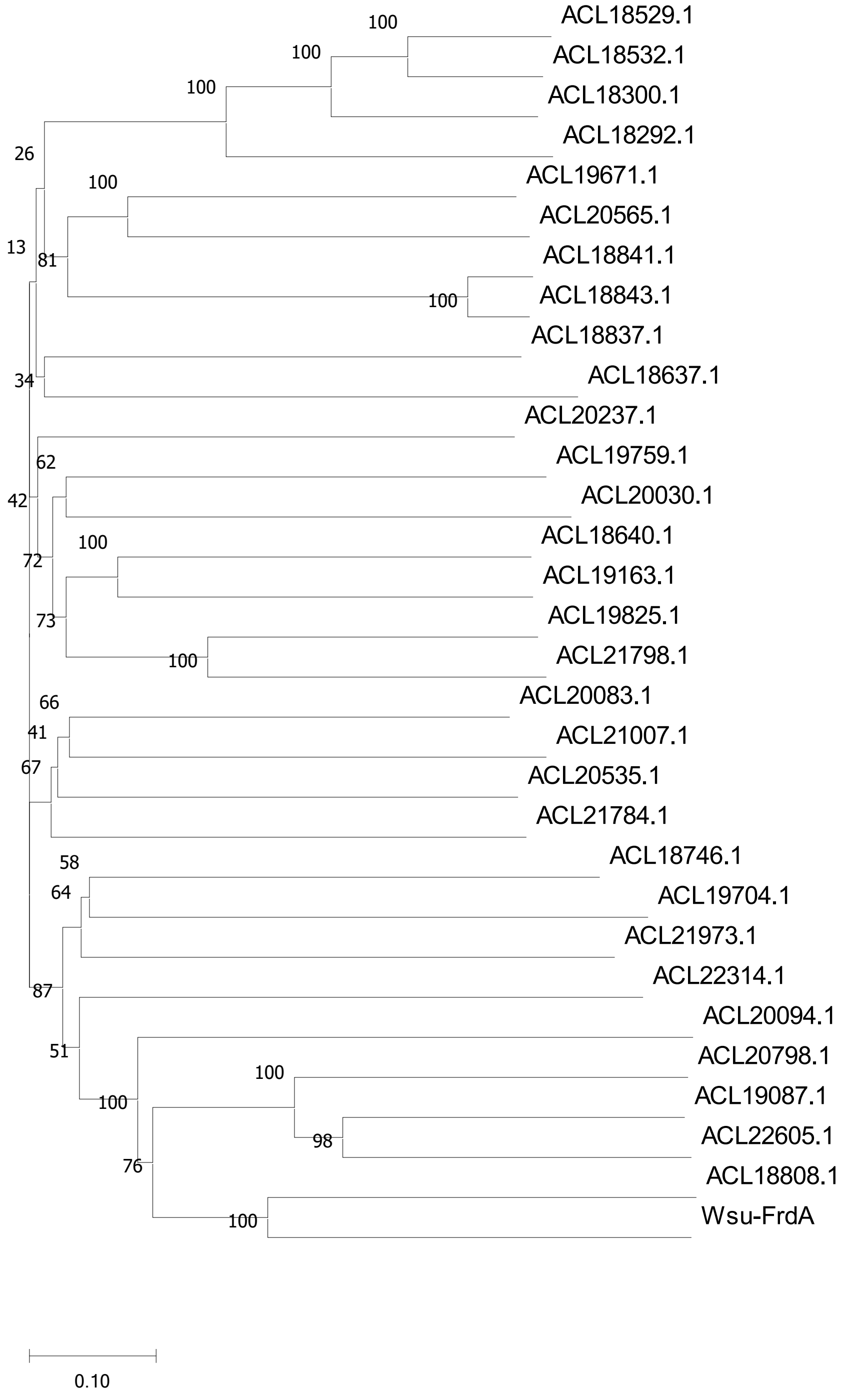

Supplementary Figure 10. Sequence likelihood analysis of the flavoprotein subunits of the succinate:quinone oxidoreductases (SQOR) from *Desulfitobacterium hafniense* strain DCB-2. The SQOR A subunits were identified in the genome of strain DCB-2 using BlastP (28), aligned including *W. succinogenes* fumarate reductase FrdA (Wsu-FrdA, P17412.3) using ClustalX2 (29) and the tree was drawn with MEGA X (30).

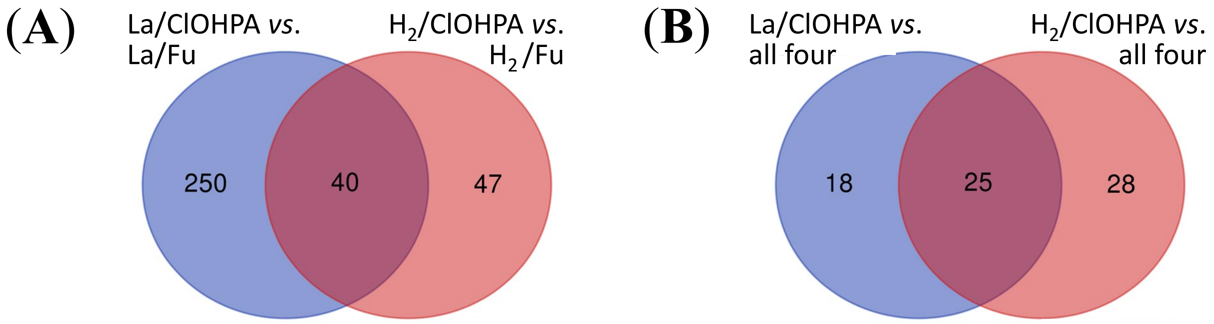

Supplementary Figure 11. Distribution of proteins that were induced by ClOHPA across different growth conditions. (**A**) Venn diagram for the distribution of up-regulated proteins as in the two pairwise comparisons depicted in the scatter plot (see Figure 6); (**B**) Venn diagram for the distribution and overlap of the 43 and 53 up-regulated proteins identified in La/ClOHPA *vs*. all non-ClOHPA and H_2/ClOHPA *vs*. all non-ClOHPA conditions, respectively.

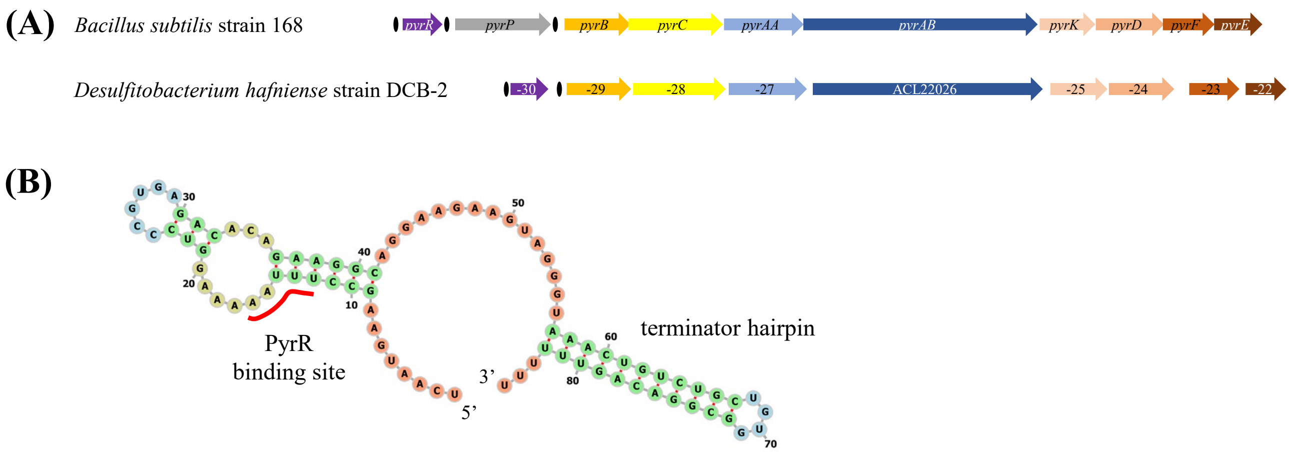

Supplementary Figure 12. Genetic analysis of the *de novo* uridine monophosphate synthesis. (**A**) Conserved gene clusters in *B. subtilis* and *Desulfitobacterium hafniense* strain DCB-2. Black ovals indicate RNA structures that act as transcriptional attenuators in *B. subtilis*. (**B**) RNAfold structure prediction of the 5'-untranslated regions of ACL22030 (*pyrR*), that displays the putative terminator hairpin and the conserved 5'-UUAAA-3' sequence motif targeted by PyrR when bound to pyrimidine nucleotides, as described for *B. subtilis*. An alternative structure forming an antiterminator hairpin (sequestering the terminator) is expected to occur when pyrimidine synthesis is needed (17).

2.2 Supplementary Tables

The contents of the Supplementary Tables are presented in a separate Excel sheet.

Supplementary Table 1. Primers used in the study.

Supplementary Table 2. Processed dataset of the overall comparative proteomic analysis.

Supplementary Table 3. Hierarchical clustering using Z-scores of proteins showing relative abundancy with at least 2× fold change in pairwise comparisons (see also Supplementary Figure 3).

Supplementary Table 4. Proteins up-regulated in Py-only condition in comparison to any of the respiratory condition.

Supplementary Table 5. Proteins displaying >10-fold up-regulated in Py-only condition in comparison to Py/Fu conditions.

Supplementary Table 6. Proteins up-regulated in H_2 conditions in comparison to lactate conditions (from scatter plot in Figure 3).

Supplementary Table 7. Proteins up-regulated in lactate conditions in comparison to H_2 conditions (from scatter plot in Figure 3).

Supplementary Table 8. Hydrogenases identified in the genome of *Desulfitobacterium hafniense* strain DCB-2.

Supplementary Table 9. Proteins up-regulated in La/Fu conditions in comparison to Py/Fu and Py-only conditions.

Supplementary Table 10. Proteins up-regulated in fumarate conditions in comparison to ClOHPA conditions (from scatter plot in Figure 6).

Supplementary Table 11. Proteins up-regulated in ClOHPA conditions in pairwise comparison to fumarate conditions (from scatter plot in Figure 6).

Supplementary Table 12. Proteins up-regulated in La/ClOHPA and H_2/ClOHPA when compared to all non-ClOHPA conditions (see also Supplementary Figure 10, E).

Supplementary Table 13. Proteins up-regulated in Py/Fu and La/Fu conditions in comparison to non-fumarate conditions.

3 References

1. Kim SH, Harzman C, Davis JK, Hutcheson R, Broderick JB, Marsh TL, et al. Genome sequence of *Desulfitobacterium hafniense* DCB-2, a Gram-positive anaerobe capable of dehalogenation and metal reduction. BMC Microbiol. 2012;12:21.

2. Bott M. Anaerobic citrate metabolism and its regulation in enterobacteria. Arch Microbiol. 1997;167(2–3):78–88.

3. Jiang F, Huang X, Barbieri NL, Logue CM, Nolan LK, Li G. Citrate utilization under anaerobic environment in *Escherichia coli* is under direct control of Fnr and indirect control of ArcA and Fnr via CitA-CitB system. Environmental Microbiology. 2021;23(3):1496–509.

4. Erb TJ, Frerichs-Revermann L, Fuchs G, Alber BE. The apparent malate synthase activity of *Rhodobacter sphaeroides* is due to two paralogous enzymes, (3S)-Malyl-coenzyme A (CoA)/beta-methylmalyl-CoA lyase and (3S)- Malyl-CoA thioesterase. J Bacteriol. 2010;192(5):1249–58.

5. Rothery RA, Workun GJ, Weiner JH. The prokaryotic complex iron–sulfur molybdoenzyme family. Biochimica et Biophysica Acta (BBA) - Biomembranes. 2008;1778(9):1897–929.

6. Zhang Y, Gladyshev VN. General trends in trace element utilization revealed by comparative genomic analyses of Co, Cu, Mo, Ni, and Se. J Biol Chem. 2010;285(5):3393–405.

7. Greening C, Biswas A, Carere CR, Jackson CJ, Taylor MC, Stott MB, et al. Genomic and metagenomic surveys of hydrogenase distribution indicate H_2 is a widely utilised energy source for microbial growth and survival. The ISME Journal. 2016;10(3):761–77.

8. Abreu IA, Lourenço AI, Xavier AV, LeGall J, Coelho AV, Matias PM, et al. A novel iron centre in the split-Soret cytochrome *c* from *Desulfovibrio desulfuricans* ATCC 27774. J Biol Inorg Chem. 2003;8(3):360–70.

9. da Silva SM, Pacheco I, Pereira IAC. Electron transfer between periplasmic formate dehydrogenase and cytochromes *c* in *Desulfovibrio desulfuricans* ATCC 27774. J Biol Inorg Chem. 2012;17(5):831–8.

10. Hägerhäll C. Succinate: quinone oxidoreductases: Variations on a conserved theme. Biochimica et Biophysica Acta (BBA) - Bioenergetics. 1997;1320(2):107–41.

11. Neale AD, Scopes RK, Kelly JM, Wettenhall REH. The two alcohol dehydrogenases of *Zymomonas mobilis*. European Journal of Biochemistry. 1986;154(1):119–24.

12. Galinina N, Lasa Z, Strazdina I, Rutkis R, Kalnenieks U. Effect of ADH II deficiency on the intracellular redox homeostasis in *Zymomonas mobilis*. The Scientific World Journal. 2012;2012:e742610.

13. Hartmann T, Leimkühler S. The oxygen-tolerant and NAD^+-dependent formate dehydrogenase from *Rhodobacter capsulatus* is able to catalyze the reduction of CO_2 to formate. The FEBS Journal. 2013;280(23):6083–96.

14. Mayer-Blackwell K, Holly Sewell, Fincker Maeva, Spormann Alfred M. Comparative physiology of organohalide-respiring bacteria. In: Organohalide-respiring bacteria. Adrian, Lorenz and Löffler Frank E. Berlin Heidelberg: Springer-Verlag; 2016. p. 259–280.

15. Studenik S, Vogel M, Diekert G. Characterization of an *O*-demethylase of *Desulfitobacterium hafniense* DCB-2. Journal of Bacteriology. 2012;194(13):3317–26.

16. Ticak T, Kountz DJ, Girosky KE, Krzycki JA, Ferguson DJ. A nonpyrrolysine member of the widely distributed trimethylamine methyltransferase family is a glycine betaine methyltransferase. PNAS. 2014;111(43):E4668–76.

17. TurnboughJr CL, Switzer RL. Regulation of pyrimidine biosynthetic gene expression in bacteria: repression without repressors. Microbiology and Molecular Biology Reviews. 2008;72:266–300.

18. Prat L, Maillard J, Rohrbach-Brandt E, Holliger C. An unusual tandem-domain rhodanese harbouring two active sites identified in *Desulfitobacterium hafniense*. FEBS Journal. 2012;279(15):2754–67.

19. Duret A, Holliger C, Maillard J. The physiological opportunism of *Desulfitobacterium hafniense* strain TCE1 towards organohalide respiration with tetrachloroethene. Applied and Environmental Microbiology. 2012;78(17):6121–7.

20. Kublik A, Deobald D, Hartwig S, Schiffmann CL, Andrades A, Bergen M von, et al. Identification of a multi-protein reductive dehalogenase complex in *Dehalococcoides mccartyi* strain CBDB1 suggests a protein-dependent respiratory electron transport chain obviating quinone involvement. Environmental Microbiology. 2016;18(9):3044–56.

21. Maillard J, Willemin MS. Regulation of organohalide respiration. In: Poole RK, editor. Advances in Microbial Physiology. Academic Press; 2019. p. 191–238. (Advances in Microbial Physiology; vol. 74).

22. Prat L, Maillard J, Grimaud R, Holliger C. Physiological adaptation of *Desulfitobacterium hafniense* strain TCE1 to tetrachloroethene respiration. Applied and Environmental Microbiology. 2011;77(11):3853–9.

23. Lancaster CRD. The di-heme family of respiratory complex II enzymes. Biochimica et Biophysica Acta (BBA) - Bioenergetics. 2013;1827(5):679–87.

24. Kruse T, van de Pas BA, Atteia A, Krab K, Hagen WR, Goodwin L, et al. Genomic, proteomic, and biochemical analysis of the organohalide respiratory pathway in *Desulfitobacterium dehalogenans*. J Bacteriol. 2015;197(5):893–904.

25. Bogachev AV, Bertsova YV, Bloch DA, Verkhovsky MI. Urocanate reductase: identification of a novel anaerobic respiratory pathway in *Shewanella oneidensis* MR-1. Molecular Microbiology. 2012;86(6):1452–63.

26. Bogachev AV, Baykov AA, Bertsova YV. Flavin transferase: the maturation factor of flavin-containing oxidoreductases. Biochemical Society Transactions. 2018;46(5):1161–9.

27. Seedorf H, Fricke WF, Veith B, Brüggemann H, Liesegang H, Strittmatter A, et al. The genome of *Clostridium kluyveri*, a strict anaerobe with unique metabolic features. PNAS. 2008;105(6):2128–33.

28. Altschul SF, Gish W, Miller W, Myers EW, Lipman DJ. Basic local alignment search tool. Journal of Molecular Biology. 1990;215(3):403–10.

29. Larkin MA, Blackshields G, Brown NP, Chenna R, McGettigan PA, McWilliam H, et al. Clustal W and Clustal X version 2.0. Bioinformatics. 2007;23(21):2947–8.

30. Kumar S, Stecher G, Li M, Knyaz C, Tamura K. MEGA X: molecular evolutionary genetics analysis across computing platforms. Molecular Biology and Evolution. 2018;35(6):1547–9.
